# Review of Filters for Air Sampling and Chemical Analysis in Mining Workplaces

**DOI:** 10.3390/min12101314

**Published:** 2022-10-18

**Authors:** Judith C. Chow, John G. Watson, Xiaoliang Wang, Behrooz Abbasi, Wm. Randolph Reed, David Parks

**Affiliations:** 1 Division of Atmospheric Sciences, Desert Research Institute, Reno, NV 89511, USA; 2 Department of Mining and Metallurgical Engineering, University of Nevada, Reno, NV 89557, USA; 3 Office of the Director, National Institute for Occupational Safety and Health, Pittsburgh, PA 15236, USA; 4 Spokane Mining Research Division, National Institute for Occupational Safety and Health, Spokane, WA 99207, USA

**Keywords:** filter, respirable coal mine dust, respirable crystalline silica, personal dust monitor, FTIR, Raman, chemical speciation

## Abstract

This review considers the use of filters to sample air in mining workplace environments for dust concentration measurement and subsequent analysis of hazardous contaminants, especially respirable crystalline silica (RCS) on filters compatible with wearable personal dust monitors (PDM). The review summarizes filter vendors, sizes, costs, chemical and physical properties, and information available on filter modeling, laboratory testing, and field performance. Filter media testing and selection should consider the characteristics required for mass by gravimetry in addition to RCS quantification by Fourier-transform infrared (FTIR) or Raman spectroscopic analysis. For mass determination, the filters need to have high filtration efficiency (≥99% for the most penetrable particle sizes) and a reasonable pressure drop (up to 16.7 kPa) to accommodate high dust loading. Additional requirements include: negligible uptake of water vapor and gaseous volatile compounds; adequate particle adhesion as a function of particle loading; sufficient particle loading capacity to form a stable particle deposit layer during sampling in wet and dusty environments; mechanical strength to withstand vibrations and pressure drops across the filter; and appropriate filter mass compatible with the tapered element oscillating microbalance. FTIR and Raman measurements require filters to be free of spectral interference. Furthermore, because the irradiated area does not completely cover the sample deposit, particles should be uniformly deposited on the filter.

## 1. Introduction

Filters are used to remove solid and liquid particles from air. The most widespread application is for air purification. Most heating and ventilation systems have some sort of filter that is regularly changed, as do air intakes on mobile and stationary engines. The earliest record of air filtration dates back to Roman times related to dust in Egyptian mines. Loose bladders were used to prevent dust inhalation and minimize health risks [1]. Use of filtering respirators [2] started in the 1800s for the removal of airborne microorganisms [3]. Early applications also included filters to protect firefighters from smoke inhalation and wetted fabrics or gas masks as protection from fumes derived from chemical warfare [4].

Filtration science was systematized and advanced in the mid-1960s by Fuchs [5] and Spurný [6] and later by Davies [7,8], who documented the history, development, and application of aerosol filtration. Filtration by Brownian motion of small particles and by impaction and interception of large particles was formulated by Langmuir [9,10] and Kaufman [11]. Later refinements applied 3D computer modeling and laboratory filter tests that revealed differences between the structures of fiber and membrane filters [1,12].

Aerosol filtration has been applied in personal protective equipment (PPE, www.who.int, accessed on 1 October 2022), industrial hygiene [13], occupational safety and health (www.osha.gov, accessed on 1 October 2022), liquid purification (by sterilization of heat-sensitive materials) [14], microplastic extraction [15,16], bioaerosols [17,18], pathogen and microorganism removal [19,20], electrospun nanofiber filtration [21], indoor air filtration, vehicle exhaust filtration, and industrial emission controls (removing particles from process gas streams). These applications emphasize particle removal rather than particle characterization. Particle characterization imposes additional constraints on the filter media in terms of blank levels, distributions across the filter surface and within the filter thickness, resistance and acquiescence to chemical extraction, and transparency to electromagnetic probes.

This review considers the use of filters to sample air in mining workplace environments for subsequent analysis of hazardous contaminants. Objectives are to: (1) survey and critically evaluate information on filters amenable to particulate matter (PM) mass and further laboratory analysis, with an emphasis on quantifying respirable crystalline silica (RCS) on filters compatible with wearable workplace dust monitors; (2) summarize filter vendors, sizes, costs, chemical and physical properties, and information available on filter modeling, laboratory testing, and field performance; (3) identify knowledge gaps and methods to fill them; and (4) recommend filter media suitable for both mass measurement by a personal dust monitor (PDM) and RCS quantification by Fourier-transform infrared (FTIR) and/or Raman spectroscopy analyses.

Several reviews of filter media for aerosol characterization have been published in the past [22–29], and these were examined first. While containing useful information, these reviews were found to be dated. Many of the manufacturers, vendors, and filter materials have changed since their publication, and more recent resources on filter characteristics and specific applications are available. This review intends to update the knowledge base with a specific focus on workplace applications in mining environments.

## 2. Ambient and Workplace Aerosol Sampling and Analysis

PM mass concentrations are used as indicators of human exposure in both ambient air and workplace environments. Samplers typically consist of a size-selective inlet, such as an impactor or cyclone, a filter holder, a collection filter through which air is drawn and onto which the PM is deposited, a pump or fan that moves the air, and a flow controller that maintains a constant volumetric sampling rate [30]. These samplers are operated for a set period of time, 24 h for ambient air and a normal work shift (8 to 12 h) for workplace monitoring. Filters are weighed before and after sampling [31], with the difference in weights divided by the flow rate times the sample duration to provide PM concentrations in μg/m^3^ or mg/m^3^. Continuous in situ methods used are: (1) filter tapes that advance when loaded with detection by beta-particle attenuation [32]; (2) replaceable filters that are continuously sensed by an inertial microbalance [33]; (3) counting of particles in different size ranges based on individual particle light scattering [34]; or (4) light scattering by an ensemble of particles calibrated against an aerosol mixture similar to that of the monitored environment [35,36].

Ambient air is sampled at fixed outdoor locations for comparison with the U.S. Environmental Protection Agency’s National Ambient Air Quality Standards (NAAQS), while workplace monitoring intends to determine individual exposure using wearable devices. Ambient PM monitoring networks [37,38] also include a subset of chemical speciation monitors for elements, soluble ions, and carbon fractions that are used to quantify source contributions, evaluate effects of PM components on visibility and health, and track air quality improvements with emission reduction strategies. Owing to the need for such characterization, a large body of information is available for ambient PM filter sampling and analysis that is relevant to the workplace. There is little information in the ambient PM literature on RCS, however, and this review provides greater scrutiny of filter media amenable to RCS analyses.

The Occupational Safety and Health Administration (OSHA, created in 1970) and Mine Safety and Health Administration (MSHA, established in 1977) develop and enforce workplace safety and health regulations. MSHA programs and activities are specifically related to mining, while OSHA addresses a broader range of workplace environments. Both agencies intend to reduce mortality, injuries, and morbidity by monitoring worker inhalation exposure. Figure S1 (Supplemental Material) shows examples of mine rescue breathing equipment that has evolved over time to meet workplace safety needs. In particular, inhalation of coal mine dust has been associated with underground coal miners’ pneumoconiosis and silicosis and is a motivation for exposure reduction.

To determine workers’ exposure to silica-bearing dust in underground and surfacemines, the MSHA established methods for coal mine sampling [39]. Table 1 summarizes the specifications for two MSHA-approved coal mine sampling devices: the coal mine dust personal sampler unit (CMDPSU) and the continuous personal dust monitor (PDM3700), as shown in Figures S2 and S3, respectively. Both devices operate at low volumetric flow rates (2.0–2.2 L/min) to measure respirable coal mine dust (RCMD, <4 μm aerodynamic diameter, also termed PM_4.0_) under local temperature and pressure conditions [40]. CMDPSU samples are acquired with filter cartridges that are weighed before and after personal sampling over a mining work shift [31].

The PDM is a tapered element oscillating microbalance (TEOM) [33,41], a miniaturized version of the ambient sampling system [42] that is a Federal Equivalent Method (FEM) for determining compliance with the 24 h PM_10_ (particles with aerodynamic diameters < 10 μm) NAAQS of 150 μg/m^3^ (not to be exceeded more than once per year over three years). The PDM3700 (Figure S3) measures RCMD mass concentrations in real time for compliance with the regulatory mining exposure limit of 1.5 mg/m^3^ over a full working shift. It is designed for mining applications with a worker-wearable (2 kg) system and PC-based software data retrieval.

Laboratory and field performance tests have found individual PDM increments within ±25% of reference sampler measurements for mass loadings between 0.5 and 4 mg/m^3^. Coefficients of variation (CV) for collocated PDMs ranged from 4.2% for concentrations between 1.5 and 2 mg/m^3^ to 6.0% for lower concentrations between 0.2 and 0.5 mg/m^3^ [43–45]. The largest paired comparison (*n* = 955) between CMDPSUs and PDMs in coal mines showed no significant statistical differences between the two systems [46]. In-mine testing produced linear relationships between CMDPSU and PDM mass concentrations [47].

Pairwise evaluations of PDM3700s with cyclone samplers in real-world mines by Belle [48] found ~50%–60% higher gravimetric mass concentration for the personal dust monitors. The large “measurement bias” between PDM3700 and gravimetric samples using a Higgins-Dewell (HD) cyclone [49] needs to be further investigated. Large variations in measured respirable dust concentrations were also reported by Verpaele and Jouret [50], who attributed the differences to ~50% oversampling by the SKC conductive black plastic sampler. These levels are double the ±25% comparability with the reference method specified by the manufacturer and reported by Belle [48]. The sampling effectiveness of different size-selective inlets [51] and the effects of sampler surfaces on particle collection should be considered when selecting appropriate filter media.

In addition to mass concentrations by gravimetry, the MSHA analyzes ~15,000 to 20,000 filter samples per year for RCS by FTIR spectrometry to enforce the RCMD standard [52]. RCS FTIR procedures are specified by MSHA [53] method P-7 and NIOSH [54] method 7603. Method P-7 includes ashing of the exposed polyvinyl chloride (PVC) filters and redepositing of the remainder onto PVC-acrylic copolymer membrane filters (DM-450 or DM-800) by isopropanol suspension prior to FTIR analysis. RCS mass is adjusted for interference from kaolinite, which is sometimes present in mine dust. The ashing and residue redepositing may introduce operational errors, but it intends to eliminate organic materials in both the coal dust deposit and the filters that might interfere with the FTIR spectra [29,55]. Additional measurement uncertainties may also result from inhomogeneities of the redeposited material on the new filter [52,56], as the FTIR beam is directed through only a section of the filter. To expedite RCS quantification, NIOSH has been developing a field-based RCS monitoring approach using a portable FTIR that can provide end-of-shift measurements on mining sites [57–63]. The fibrous filter mat in the PDM3700 consists of borosilicate fiberglass with a polytetrafluoroethylene (PTFE) polymer binder cured at 370 °C and backed with a woven glass fiber support (EMFAB™ TX40HI20WW, Pall Laboratory) that interferes with RCS quantification by FTIR and Raman spectroscopy. The PDM3700 filter holder is not designed to accommodate filter media with chemical stability and low absorbance for spectroscopic determination of chemical components, including RCS. Because the current PDM filter assembly is not amenable to RCS quantification [29,64], the CMDPSU with PVC filters is also needed for field-based FTIR analysis. Reduced sampling effort and increased monitoring efficiency can be achieved when both RCMD and RCS concentrations can be determined from the PDM filter.

## 3. Filter Characteristics

Table S1 summarizes chemical and physical characteristics of 12 filter types along with compatible physical and chemical analyses. The 11 vendors identified in Table S1 provide multiple filter media types and it is not clear that the vendors are also media manufacturers. When contacted for this review, vendors were not forthcoming on filter origins for proprietary reasons. To achieve mass closure with measured chemical components [65], it is often necessary to sample concurrently on multiple substrates.

Air sampling filters vary in material, structure, filter diameter, pore size, thickness, mechanical and temperature stability, chemical compatibility, blank concentrations, flow resistance, particle loading capacity, and collection efficiency [27]. With the exception of the fluorinated ethylene propylene (FEP) membrane film used as an impaction surface for cascade impactor sampling, filters are porous structures that accommodate different flow rates, flow pathways, residence times, and applications.

Table S1 categorizes the main classes of filters that are commonly used for aerosol sampling, including: (1) six types of membrane (also termed “porous membrane”) filters (i.e., PTFE, polypropylene, PVC, nylon, silver, and mixed cellulose esters [MCE]); (2) one type of capillary pore filter (i.e., polycarbonate); and (3) five types of fibrous filters (i.e., cellulose fiber, pure and mixed quartz fiber, Teflon-coated glass fiber, and glass fiber) [26,66]. The following sections: (1) describe materials and structures of these filter types; (2) summarize past tests on filter collection efficiency; (3) discuss potential atmospheric artifacts; (4) examine effects of particle deposit inhomogeneities; and (5) tabulate filter costs and availability.

### 3.1. Filter Material and Structure

Figure S4 [67] compares the structures among porous membrane, capillary pore, and fibrous filters, showing different surface topographies. The largest contrast is found between glass- and quartz-fiber filters with randomly crossed fibers and those with uniform passages in the capillary pore filter. Porous membrane filters are gels formed from a colloidal solution having interconnected pores with uniform microstructures that capture particles on the filter surface while allowing the passage of air through the filter volume. They consist of different synthetic materials, including: (1) PTFE membrane, a microporous membrane made of a synthetic fluoropolymer of PTFE; (2) polypropylene, also known as polypropene, a thermoplastic polymer produced by chain-growth polymerization from monomer propylene; (3) PVC membrane, produced by free-radical polymerization of PVC, a polymer similar to polyethylene but with one of the hydrogen atoms replaced by chlorine atoms; (4) nylon membrane, made of diacid chlorides, diamines, polyamide, or thermoplastic polymers; (5) silver membrane, consisting of sintered pure metallic silver (~99.97%); and (6) MCE membrane, made of different cellulose molecules containing hydrocarbon polymers (e.g., carbon, hydrogen, and β-glucose). MCE is a mixture of cellulose esters, cellulose acetate, and cellulose nitrate with compositions varying among manufacturers. Most membrane filter disks are thin films (~30–70 μm) manufactured with various pore sizes (0.2–5 μm). These filters have adequate porosity (>85%) for high particle collection efficiency, but they require powerful flow movers to overcome resistance across the filter. Smaller pores usually require larger pumps for a given flow rate.

After the promulgation of the PM_2.5_ (particulate matter with aerodynamic diameter < 2.5 μm) NAAQS [68], 37 and 47 mm-diameter PTFE thin-film membrane filters stretched across a support ring (e.g., polymethylpentene [PMP] and FEP with polypropylene) have become the most commonly used substrates for gravimetric analysis [31,69]. These ringed-membrane filters are used in U.S. PM_2.5_ compliance and speciation networks and for elemental analysis [70] in addition to mass concentration. Nylon membrane filters with upstream denuders are used in a separate channel for sampling and analysis [71] of major ionic species, such as sulfate (SO_4_^2−^), nitrate (NO_3_^−^), and ammonium (NH_4_^+^), in speciation networks [72] as they minimize the loss of semivolatile ammonium nitrate (NH_4_NO_3_) during and after sampling [73]. Pure quartz-fiber filters are used to measure carbonaceous aerosols [74–76] because their composition is carbon-free.

Capillary pore filters consist of polycarbonate with polyester. They are manufactured from a polycarbonate sheet in contact with uranium (U) in a nuclear reactor. The neutron flux from U-235 fission creates uniform holes in the plastic [66] that are perpendicular to the filter surface. These holes are acid-etched for different durations to obtain a wide variety of pore sizes. During the early 1980s, large-pore (~8 μm) Nuclepore polycarbonate membrane filters were used as size-selective inlets in the stacked filter unit (SFU) [77] for sampling PM of different size fractions in the U.S. National Parks; smaller particles passed through the pores while larger particles remained on the filter surface. Polycarbonate membrane filters have low porosity (5%–10%) with adequate collection efficiency, depending on pore size. These filters are commonly used for bioaerosol sampling and image processing [17,18,20]. They are especially amenable to automated single particle analyses for particle size, shape, and color using scanning electron microscopy (SEM) [78], transmission electron microscopy (TEM), and/or optical microscopy, as pattern recognition techniques can separate the pores from the particles. These filters rapidly acquire static charges [79–81] that must be neutralized prior to gravimetric analysis.

Fibrous filters are composed of a mat or weave of randomly oriented individual fibers. They are thicker than membrane filters (~200–500 μm) with various porosities (60%–90%) and sampling efficiency. Glass- and quartz-fiber filters were commonly used for high-volume air sampling during the 1960s–1990s [27]. These filters are suitable for limited elemental [70,82,83], ionic [71,84], and carbon [85–89] speciation, depending on the analysis method and filter purity. Mixed quartz-fiber filters consist of quartz fibers with ~5% borosilicate that has a lower (~500 °C) melting point, causing measurement uncertainties for carbon analysis and contributing some trace elements to the blank. Pure quartz-fiber filters have high temperature resistance, but they are brittle and portions of their edges may become attached to the filter holder or flake off, thereby negatively biasing mass measurements. Ultrapure quartz-fiber filters are used in the U.S. long-term Interagency Monitoring of Protected Visual Environments (IMPROVE) network and Chemical Speciation Network (CSN) for carbon analysis [74,76,85]. These filters adsorb organic vapors [90,91], and field blanks or backup filters are needed to correct for this organic carbon bias.

Cellulose-fiber filters are made of a thick layer of paper fibers. As this material is carbon-based, these filters are inappropriate for carbon analysis. Cellulose-fiber filters have low and variable filtration efficiency (~70%) [92] and they absorb water, which can cause filter-weighing biases unless the balance environment maintains a constant relative humidity (RH) [93]. These filters are best used for absorbing gases after being impregnated with acid or base solutions. The impregnated filters can be placed behind a Teflon-membrane or quartz-fiber filter to capture precursor gases, such as sulfur dioxide (SO_2_), nitrogen dioxide (NO_2_), nitric acid (HNO_3_), and ammonia (NH_3_) [94,95]. Citric acid, oxalic acid, and phosphoric acid have been used for sampling of NH_3_, while sodium carbonate and potassium carbonate have been used for collecting SO_2_ and organic acids [96]. Triethanolamine (TEA) has been used as an absorbent for collecting NO_2_, peroxyacetyl nitrate (PAN), organic nitrates, and SO_2_.

Most filters in Table S1 list a pore size. While the capillary pore filters have well-defined pore diameters, membrane and fibrous filters do not have simple pore structures. For these filters, the pore size refers to an “equivalent pore diameter,” which is determined by a bubble-point test [97]. For the same type of filter, filtration efficiency and pressure drop vary systematically with pore size. However, filtration characteristics for the same pore sizes of different filter types are not expected to be the same [98].

Few manufacturers listed in Table S1 provide filter ash contents. As noted, MSHA [53] method P-7 ashes PVC filters prior to FTIR analysis, so this is an important specification. Ash content is expressed as a percentage of the original filter mass remaining after the filter is baked at temperatures > 500 °C (typically 800 °C). Low ash contents are desired when heat-resistant minerals such as quartz are to be isolated from the collection substrate. Carbon-containing filters have low ash contents as the carbon is easily combustible, while the glass- and quartz-fiber filters have high ash contents. Lippmann [66] summarized ash contents for a variety of filters, reporting values on the order of <0.001% for MCE and 0.01% each for cellulose-fiber and polycarbonate filters. Ash contents are not reported for Teflon-, nylon-, cellulose acetate-, cellulose nitrate-, PVC-, or silver membrane- filters. The glass- and quartz-fiber filters have ash contents > 95%, as expected owing to their mineral compositions.

### 3.2. Filter Collection Efficiency

**Figure 1.**
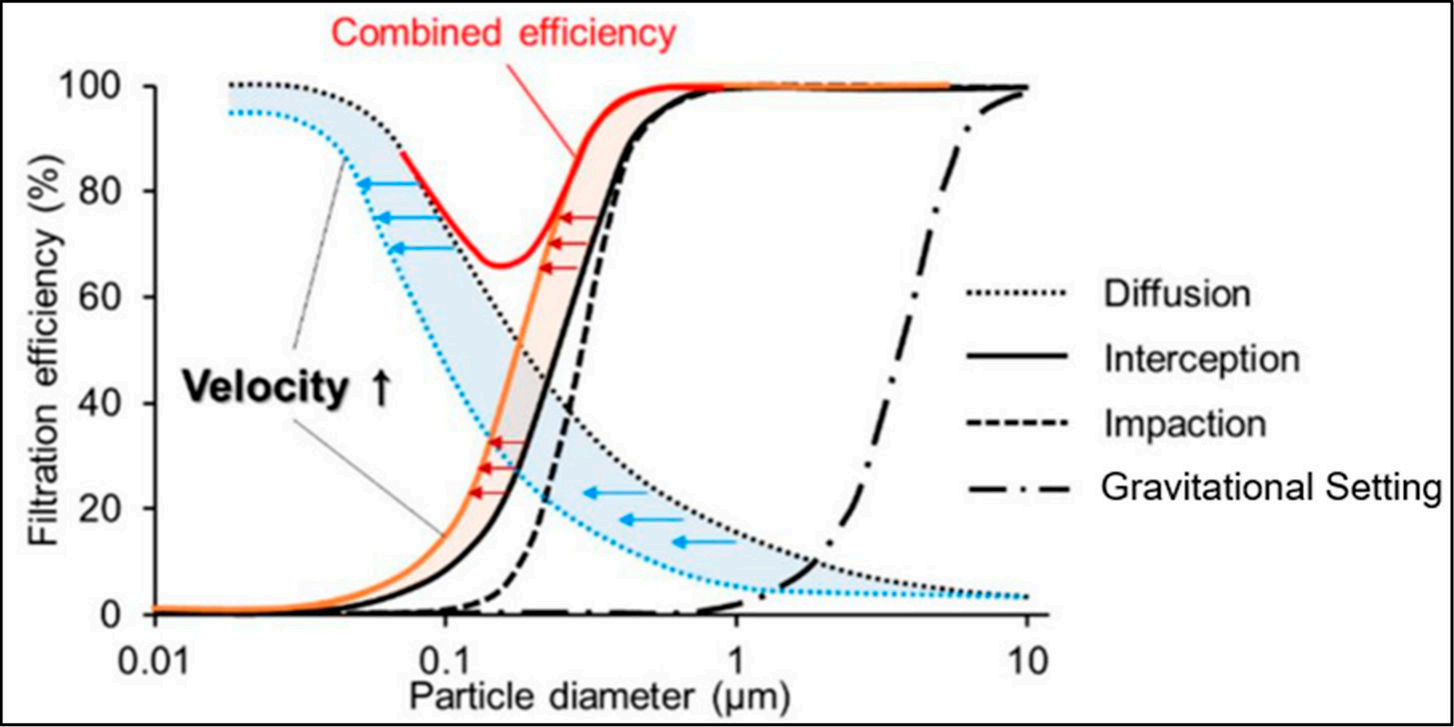
Illustration of the combined effects of the particle capture mechanisms for solid particles on the overall filtration efficiency [12].

Except for selective filtration using large-pore polycarbonate filters, air sampling filters should capture more than 99% of suspended particles, regardless of particle size or flow rate [66]. Membrane filters have been used in air sampling for over 60 years [99–101]. Lower porosities and pore sizes generally result in higher sampling efficiency but increases in flow resistance. The filtration process includes a variety of collection mechanisms that alter the filter collection efficiency for various particle sizes under different sampling face velocities, which are assumed to reflect the particle velocities [22]. Decreases in particle size enhance particle collection by Brownian motion, whereas increases in particle size lead to an increase in filtration by interception, inertial impaction, and gravitational settling [12,21], as illustrated in Figure 1. Particle collection efficiency is usually at a minimum for particles with ~0.3 μm aerodynamic diameters [102–104], and many filtration test methods limit themselves to this size of test aerosol.

Diffusion is the primary mechanism for collecting ultrafine particles (<0.1 μm), with higher efficiency for smaller particles. Impaction, interception, and gravitational settling are main mechanisms for collecting larger (≳0.5 μm) particles, with higher efficiency for larger particles. The combined effects in Figure 1 show that both small and larger particles have high filtration efficiency, while an intermediate size range (typically 0.05–0.5 μm) has lower efficiency, where none of the mechanisms are most effective. Filtration performance has commonly been determined in the laboratory by generating and measuring the number concentrations of monodisperse particles or the size distributions of polydisperse solid particles (e.g., sodium chloride [NaCl]) or plasticizer (e.g., 0.3 μm dioctyl phthalate [DOP] droplet) before and after the filter for selected face velocities [75].

Liu et al. [22] reports collection efficiency for 76 filters of various pore sizes tested with monodisperse particles (i.e., 0.035, 0.01, 0.30, and 1.0 μm diameters) at various pressure drops. Values relevant to CMDPSU and PDM 3700 sampling are summarized in Table 2. Filter permeability is characterized by the face velocity measured at a pressure drop of 1.3 kPa across the filter.

The CMDPSU uses a 5 μm pore PVC filter, and Table 2 shows good collection efficiency (96.7%–99.9%) for Millipore (www.emdmillipore.com, accessed on 1 October 2022) filters but with lower collection efficiency (49%–98.8%) with the more permeable Metricel (us.vwr.com, accessed on 1 October 2022) 5 μm pore PVC filters. The Metricel 0.8 μm pore PVC filter shows higher collection efficiency (99.96%–>99.99%). Different Teflon-membrane filter types (e.g., Gelman, Ghia, and Zefluor) have high collection efficiency (85%–99.99% for 2–5 μm pore sizes). Collection efficiency for silver membrane filters increases with decreasing pore size. For 5 μm pore filters, efficiency is as low as 25% for smaller particles, while 0.45 μm pore filters have efficiency >93.6% for all tested particle sizes.

The two lots of Teflon-coated glass-fiber filters (TX40HI20 used in the PDM3700) have collection efficiency of 92.6%–99.6% and 98.9%–99.99%, higher than the Pallflex 2500QAO quartz-fiber filters (84%–99.99%). Low collection efficiency was found for Whatman 41 (a commonly used cellulose-fiber filter) (43%–99.5%) and Whatman 40 (77%–99.99%). Filtration efficiency for glass-fiber filters is high (98.5%–99.99%). Kim et al. [105] noted that filtration efficiency for glass-fiber filters can be affected by particle charge, but independently of RH. As noted for polycarbonate, filter materials may acquire electrostatic charges that bias the mass determination unless adequately neutralized. Modern weighing facilities use ionizing blowers or polonium-210 charge neutralizers to eliminate this bias [31].

**Figure 2.**
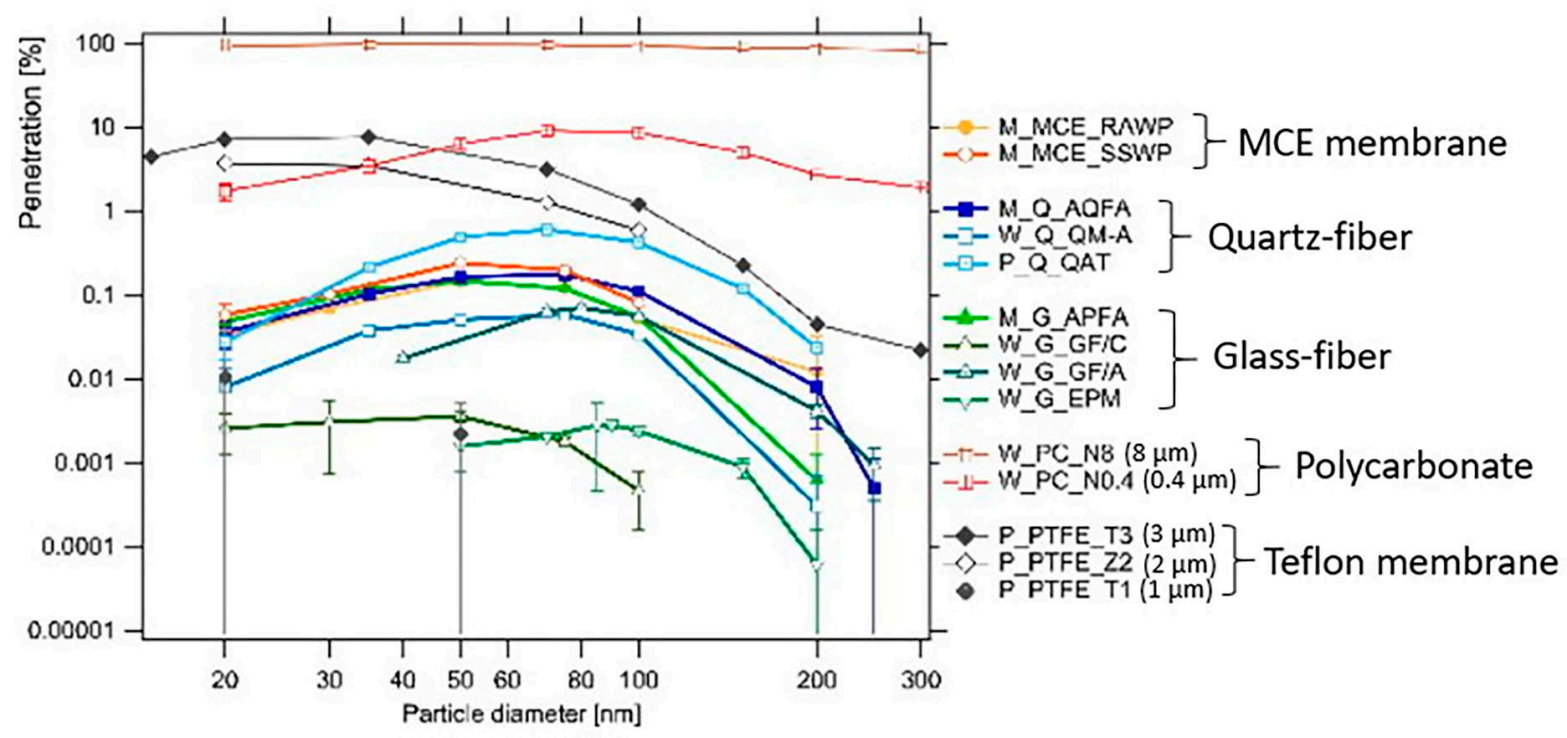
Size-resolved penetration of submicron particles by mixed cellulose esters (MCE), quartz, glass fiber, polycarbonate (PC, 0.4 and 8 μm), and Teflon (Zefluor 2 μm and Teflo 1 and 3 μm) at a face velocity of 40 cm/s [106]. Reprinted by permission of Taylor & Francis Ltd. on behalf of the American Association for Aerosol Research.

While filter efficiency determines the fraction of sampled particles that are retained on the collection media, filter penetration denotes the fraction that passes through the filter (i.e., 100% minus collection efficiency). Zíkova et al. [106] reported large variations in size-dependent penetration for ~20–300 nm diameter particles among the five filter types. Figure 2 shows the lowest penetration (0.001%–0.1%) for glass fiber, ~0.1% for MCE and quartz fiber, ~10% for PTFE Teflon filters, and the highest penetration for 0.4 and 8 μm polycarbonate filters. The percentage penetration maximum varied by four orders of magnitude among different filter media with most penetrable particle sizes in the 20–86 nm range, mostly <50 nm. As shown in Figure S5, particle penetrations vary among filter types, but to a much lesser degree for different batches of the same type of filter.

**Figure 3.**
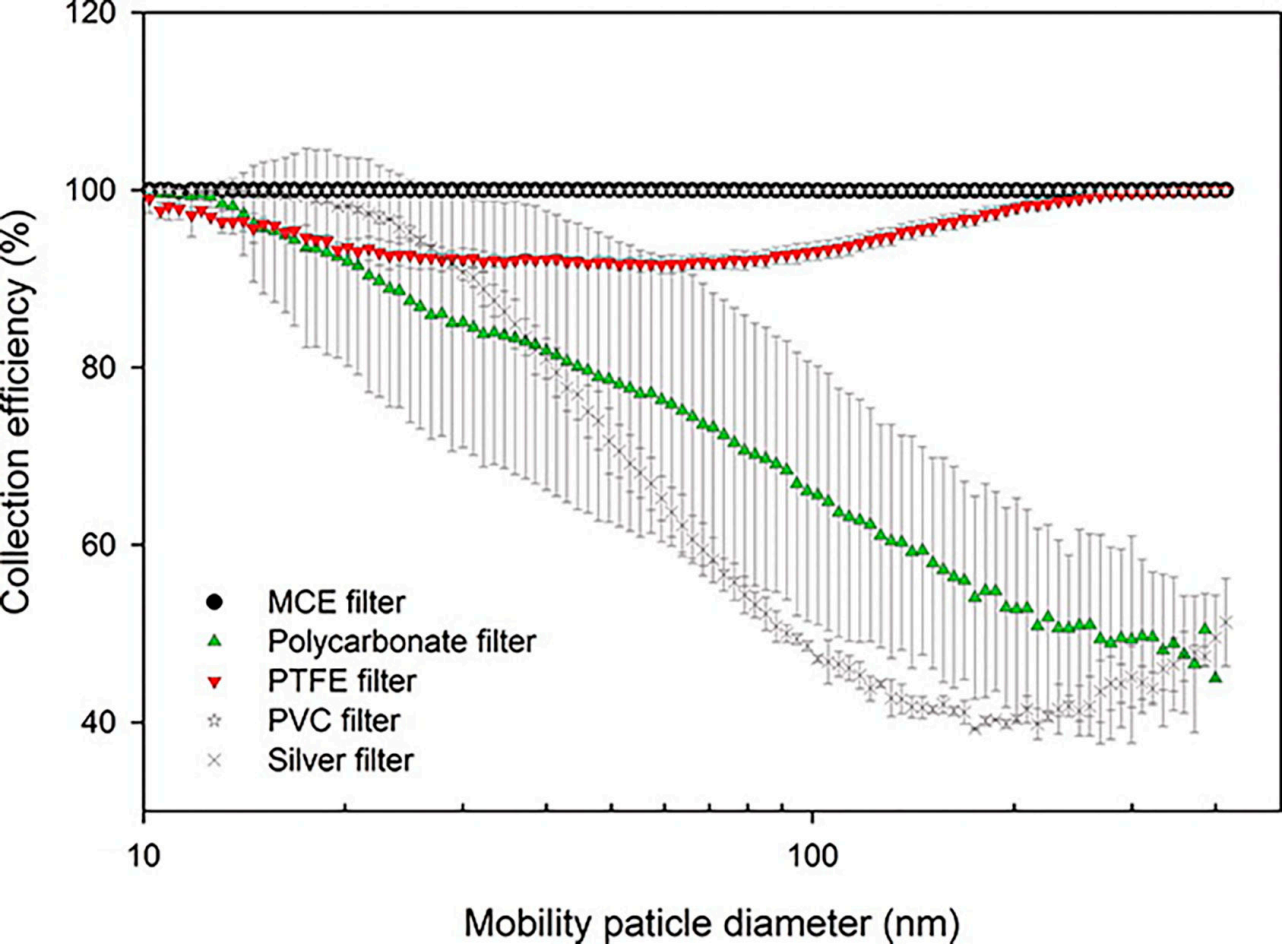
Average and standard deviation of collection efficiency of five types of 37 mm filters with 5 μm pore size tested with a nanoparticle diameter range of 10–400 nm at 1.7 L/min. Aerosol measurements were conducted using three different filters for each filter type (n = 3). The two overlapping point symbols for MCE and PVC filters are denoted as solid circle and star symbols [107]. Reprinted by permission of Taylor & Francis Ltd. on behalf of the American Association for Aerosol Research.

Figure 3 [107] compares small-particle (<300 nm) collection efficiencies for five 5 μm pore membrane filters. The highest collection efficiency was found for MCE filters with rapid and variable efficiency decreases with increasing particle size for the polycarbonate and silver membrane filters. There is a ~5%–10% reduction in PTFE collection efficiency for 20–200 nm particles. Table 3 [107] shows descriptive statistics from testing a range of pore sizes (0.4–5 μm) and flow rates (1.7–11.2 L/min) including four of the five 5 μm pore filters in Figure 3. For the range of filter pore sizes 0.45–5 μm, median collection efficiency integrated over the 10.4 to 412 nm size range of the NaCl test aerosol was lowest (96%) for silver membrane but comparable among the PTFE, PVC, and MCE membrane filters (99.7%–99.9%). Millipore 5 μm pore polycarbonate at 3.11 cm/s face velocity exhibited the lowest collection efficiency (22.48%) with large variations (85.3 ± 22.2%), consistent with that for 5 μm Nuclepore filters, with efficiency as low as 6%, reported in Table 2 [22].

Large collection efficiency variations were also found for silver membrane filters with an average and standard deviation of 86.5% ± 20.3% for a mixture of 0.45, 0.8, 1.2 and 5 μm pore sizes. The lowest collection efficiency (42.1%) was found for the 5 μm-pore Sterlitech (www.sterlitech.com, accessed on 1 October 2022) silver membrane filter tested at a face velocity of 8.06 cm/s, consistent with those reported by Liu et al. [22] with a low 25% collection efficiency for a 5 μm Flotronics filter.

Individual tests of 5 μm-pore PVC from three manufacturers (i.e., SKC, Gelman, and Millipore) yielded 92.98%–99.95% collection efficiency [107]. The SKC (www.skcinc.com, accessed on 1 October 2022) 5 μm PVC filter tested at a face velocity of 20.5 cm/s showed the lowest collection efficiency (92.98%) among the three vendors. This is comparable to the >96.7% for Millipore but much higher than the >49% for Metricel shown in Table 2. Farcas et al. [52] concluded that a 5 μm PVC filter is a suitable replacement for the DM-450 filter used for coal dust quartz determination by FTIR using the MSHA P-7 and NIOSH 7603 methods, but they noted that further experiments are needed to confirm the high collection efficiency of small particles. Overall, Soo et al. [107] confirmed the high collection efficiency (>99.7%) of PTFE, PVC, and MCE filters with higher variations found for polycarbonate and silver membrane filters for experimental particles smaller than their pore sizes and for different flow rates.

Collection efficiency and pressure drop vary with filter pore sizes [98,108]. PDMs are designed for pressure drops up to 16.7 kPa (125 mmHg) to accommodate high dust loadings [29]. For the same filter type, collection efficiency and pressure drop increase with decreasing pore size. A power-law relationship was found between the pressure drop and flow rate with higher pressure drops for small pores. Figure S6 shows large variations in initial pressure drop from 0.137 to 2.5 kPa as pore sizes decrease from 5 μm to 0.45 μm at 1.7 L/min [107]. Large increases in pressure drop were also found as flow rates increased from 1.7 to 11.2 L/min.

Collection efficiency and pressure drop change with particle loading [109]. Due to formation of a dust cake on the filter surface, the filtration efficiency will initially increase with added dust loading, especially for capillary pore filters that rely on surface interception [110]. Soo et al. [107] found collection efficiency increases for silver membrane and polycarbonate filters with increasing sample durations (sampling after 270 and 540 s) while collection efficiency for PTFE, PVC, and MCE did not show noticeable differences in particle loadings. Longer sample durations in heavily polluted environments, however, may result in filter clogging and increased pressure drop, leading to insufficient airflow through the filter [26].

The collected particles should not clog filters over a specified sampling period (e.g., 24 h or an 8 or 12 h shift). Table 1 shows that the PDM3700 can collect particle mass from 0.1 to 4 mg. Membrane filters used in samplers with low- and medium-volume inlets generally have higher flow resistance and lower loading capacity than fiber filters [13]. Lower resistance and higher capacity can be attained by increasing the filter exposed area or pore size and reducing the filter thickness. Lower flow resistance is often gained at the expense of decreased filtration efficiency.

### 3.3. Potential Environmental Artifacts

High temperature and moisture environments in underground mines may result in sampling artifacts that bias mass and chemical measurements. Filters should be chemically inert and retain their porosity and structure during sampling. However, the physical and chemical properties of filters listed in Table S1 may lead to: (1) adsorption and desorption of gases in the sampled airstream; (2) evaporation of volatile and semivolatile materials; (3) reactions with the water vapor; and (4) particle losses due to lack of adhesion to the filter surface during sampling and handling.

Gases adsorbed by the filter material result in positive biases to mass and chemical concentrations. Depending on face velocities, some particles change to gases, or volatilize, when temperatures, RH, and precursor gas concentrations change during field sampling, filter handling, transport, and storage. Volatilization causes a negative bias to mass and chemical composition and is more dependent on environmental variables than on the filter composition.

It is well known that adsorption of SO_2_ and nitrogen oxides (NO_x_) on borosilicate glass fibers results in positive sulfate and nitrate artifacts [111–115]. This was recently confirmed in laboratory and field tests by Gilbert et al. [116]. Some studies use quartz-fiber filters to collect samples from SO_2_-containing sampling streams. Ambient RH and concentrations of NH_3_ and HNO_3_ gases affect the gas–particle equilibrium of NH_4_NO_3_, but temperature is the most important variable [117]. Evaporation of NH_4_NO_3_ during warm-season sampling has been documented [73,118,119]. Febo et al. [120] found that cellulose-fiber filters retain both HNO_3_ and nitrate (positive artifact), in contrast to Teflon-membrane filters (negative artifact). Keck and Wittmaack [121,122] reported adequate sampling of NH_4_NO_3_ and ammonium chloride (NH_4_Cl) with MCE membrane filters that retained large fractions of particle evaporation. The chemical stability of the MCE needs to be further examined. Instead of using a backup filter to evaluate particle evaporation [96], Keck and Wittmaack [123] measured semivolatile inorganic PM using a denuded cellulose-fiber filter.

Adsorption of SO_2_, NO_x_, water vapor, and diesel exhaust fumes are potential interferents for mass and chemical measurements in underground coal mines. The use of nonacidic and nonalkaline filters (e.g., Teflon membrane or quartz fiber) largely eliminates these artifacts. The adsorption of organic gases by quartz-fiber filters is still an interferent for mass and organic carbon concentrations [90,91,124]. Organic gas adsorption (positive bias) may counteract organic particle volatilization (negative bias). However, as sample durations increase, the proportion of the adsorption bias decreases because the adsorbed gases reach equilibrium with the collected particles and the filter may become saturated. The composition of the organic gases and particles in the sampled air may affect the magnitude of the artifact.

During sampling, water vapor can also adsorb to or desorb from the filter. Allen et al. [125] reported large water uptake by glass-fiber filters with less RH influence for Teflon-coated glass-fiber filters in woodsmoke emissions testing. Comparison of gravimetric mass between uncontrolled (24–28.5 °C and 40%–65% RH) and controlled (22–26 °C and 39.5%–41% RH) environments by Tsai et al. [126] found a higher CV of 0.27% for MCE (Table S2). Particle mass measured on Teflon-membrane and PVC filters is more stable than measurements on glass-fiber filters, irrespective of equilibration condition. CVs for Teflon-membrane filters (0.0019%–0.002%) are an order of magnitude lower than those of PVC filters (0.016%–0.017%). The U.S. EPA regulations for PM_2.5_ mass concentration require environmental equilibration of filters within temperature (21.5 ± 1.5 °C) and RH (35% ± 5%) ranges for at least 24 h prior to gravimetric analysis [31] to minimize moisture effects.

Since the PDM vibrates the filter to determine mass concentrations, the particles must not be moved or dislodged due to the filter motion [42]. A fibrous filter is currently used with the assumption that the fibers hold the particles in place. However, particle loss due to lack of filter adhesion during vibration or filter transport has not been extensively tested for any of the filters. Under normal sampling conditions, particle bounce, in which particles deposited on a substrate are removed by collisions with incoming particles, has been observed during cascade impactor sampling. This becomes more common at higher face velocities.

MSHA method P-7 employs low temperature ashing and redeposition onto another filter before spectroscopic analysis. Because the current PDM filter holder cannot be disassembled, the entire assembly needs be ashed. Tuchman et al. [29] shows that the polypropylene filter assembly containing titanium dioxide produces a strong and broad IR absorption spectrum for wave numbers of 450–850 cm^−1^ that interferes with RCS quantification. On the other hand, the ashed clear polypropylene shows much lower absorbance by FTIR analysis. Potential interference of filter holder on RCS analysis by FTIR should be considered when designing filter holders.

### 3.4. Inhomogeneous Sample Deposits and Filter Cassette Assembly

**Figure 4.**
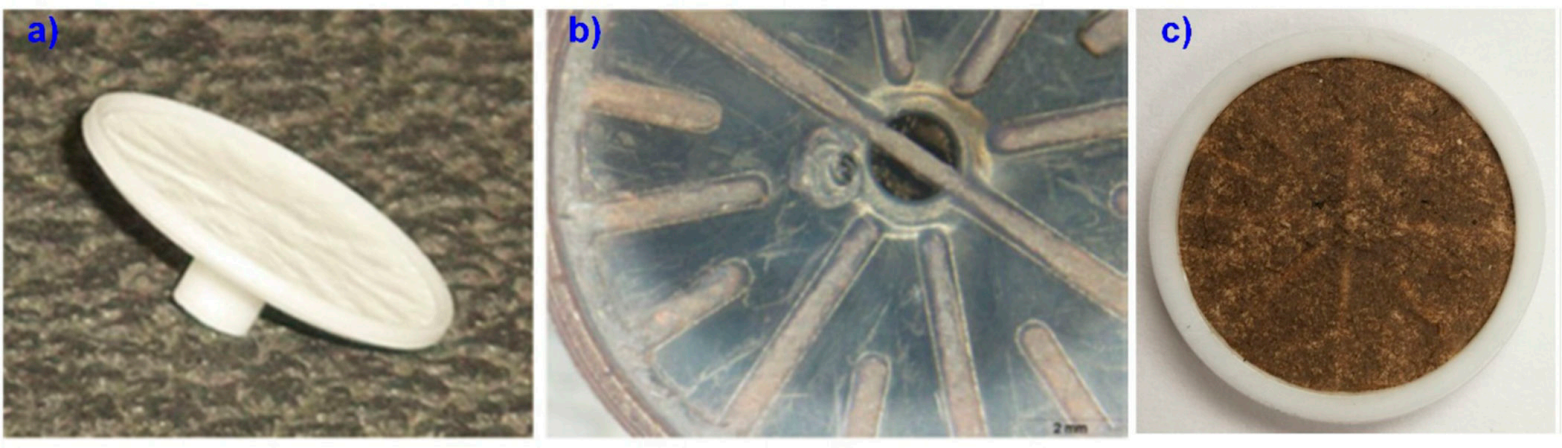
Examples of: (**a**) a clean PDM filter holder; (**b**) backside of a PDM filter holder showing structural bars; and (**c**) a PDM filter with a nonuniform particle deposit affected by the filter holder [64]. Reprinted by permission of Taylor & Francis Ltd.

Filters should lie flat on top of the filter cassette to prevent leakage. Open-faced filter holders with no upstream constrictions provide homogeneous sample deposits, but in-line filter holders without a mixing zone above the filter surface cause more particles to be collected in the filter’s center [127]. This is not an issue if the entire filter is digested and analyzed, but it biases results when only parts of the filter are examined. The PDM filter assembly (Figure 4) with a circular polypropylene base on top of a hollow axial stem results in an inhomogeneous particle deposit. Figure S7 shows an inhomogeneous sample deposit for 0.45 μm nylon filters used for redeposition of laboratory-generated coal dust compared to polypropylene and PVC [52].

Nondestructive spectroscopic analysis irradiates a small particle deposit area, then normalizes the concentration to the entire exposed area, so a uniform surface deposit is needed [27]. Miller et al. [60] used three different systems sampling laboratory-generated minusil (Min-U-Sil, a standard source of pure silica with a 92% α-quartz content) [128] and coal dust. Analyses of silica by FTIR were directed to nine different spots across each filter by moving the filter holder in 3 mm increments. CVs of 4%–51% were found, depending on the number of replicate analyses and the type of sampling device. Miller et al. [60] found that samples collected with three-piece cassettes using a single-spot FTIR analysis produced adequate CVs of ~15%. For the CMDPSU, the 37 mm 4-piece black polypropylene conductive housing (Figure S2) features additional spacer rings to increase the distance between the cyclone inlet and filter, resulting in a more homogeneous particle deposit. A redesign of 13 mm filter cassette assembly for the PDM needs to employ an open-face sampling concept to ensure homogeneous particle deposits.

### 3.5. Cost and Availability

Some of the filter media documented during the 1980s–1990s are no longer commercially available. Consistency in filter quantity and manufacturer production is needed for long-term coal mine sampling. Table S1 documents a variety of filter disks from 13 to 142 mm in diameter that accommodate different filter cassettes and sampling systems, with the most common being 37 and 47 mm. Large rectangular 20.3 cm × 25.4 cm quartz- and glass-fiber filters have been used for high-volume PM_10_ sampling [129].

Filter quantities vary between 25 and 100 per box with various pore sizes. For comparison, Table S1 separates costs into low (≤US$300 per 100 filters), medium ($301 to $500 per 100 filters), and high (>$500 per 100 filters) categories. Filter costs have increased over time with variations by filter type and pore size. The highest costs are for silver membrane filters, ranging from $7 to $20 per filter, and PTFE Teflon-membrane filters, ranging from ~$5 to $12 per filter. Nylon membrane filter costs are low (~$2 to $3 per filter). The lowest cost is found for polycarbonate, cellulose-fiber, and glass-fiber filters in the range of $1 to $1.50 per filter.

To accommodate PDM3700 sampling, 13 mm-diameter filters are only available from three vendors for Teflon, silver membrane, and polycarbonate filters with various pore sizes. These include: (1) Pall Corporation (us.VWR.com, accessed on 1 October 2022) TF filter (0.45 μm); (2) SKC (www.skcinc.com, accessed on 1 October 2022) PTFE (5 μm); (3) SKC silver membrane (0.2, 0.45, 0.8, 1, 2, 3, and 5 μm); (4) Sterlitech Corporation (www.sterlitech.com, accessed on 1 October 2022) silver membrane (0.2, 0.45, 0.8, 1.2, 3, and 5 μm); and (5) Sterlitech polycarbonate (3 μm). These 13 mm filters are ~$3 to $4 per filter. Some filters without commercially available 13 mm sizes can be punched from larger sizes to produce 13 mm disks.

## 4. Membrane Filters for Respirable Crystalline Silica (RCS) Quantification

Silica, or silicon dioxide (SiO_2_), is present in both crystalline and amorphous (polymorphic) forms. Crystalline silica is a mineral commonly found in the forms of sand, soil, stone, brick, and concrete, whereas RCS is mechanically generated when cutting, clipping, grinding, drilling, sanding, sawing, and crusting rocks and stones that present health risks in workplaces.

Table S3 shows three major infrared bands of crystalline silica, including α-quartz, cristobalite, and tridymite. Of these, quartz is a naturally occurring crystalline mineral that consists primarily of silica with some impurities in α-quartz, a low-temperature phase quartz that is the most abundant and thermodynamically stable form of crystalline silica. Cristobalite and tridymite are rare: they can occur in igneous rocks, but generally in very small amounts. Tridymite can also occur in highly metamorphosed impure limestones and arkoses, and there can be trace amounts of cristobalite in soils [130]. Other crystalline silica minerals include keatite, coesite, stishovite, and moganite. Coesite and stishovite are rare in nature. Keatite is a synthetic form not normally found in nature [130]. Published literature uses the terms silica, quartz, α-quartz, crystalline silica, and RCS interchangeably. OSHA established standards to protect workers’ exposure to RCS (29 CFR 1926.1153). The following sections discuss spectroscopic methods, potential interference, and detection limits for RCS analysis by FTIR and Raman spectrometry.

### 4.1. Spectroscopic Analysis Methods for Coal Mine Dust

The most common analytical methods for determining RCS in dust samples are infrared (IR) spectrometry and X-ray diffraction (XRD) [59,131–134]. Currently, IR is used for RCS analysis in coal dust [53,54], while XRD is used for RCS analysis in metal and nonmetal mining dust [54,135]. XRD determines the structure and composition of crystalline substances in dust and rocks semiquantitatively. It is less affected by interference by distinguishing different types of crystalline silica (e.g., quartz, cristobalite, and tridymite). However, XRD analysis is labor intensive and not commonly applied to filter samples compared to the IR or FTIR methods. IR spectrometry is less costly with good precision [57,61], but it is also a less specific chemical characterization technique. Comparisons between XRD and IR spectrometry for the analysis of α-quartz show agreement on average (within 2%) with large variabilities (with differences up to 41.3%) in individual sample comparisons [132].

#### 4.1.1. FTIR Spectrometry

FTIR spectrometry has been applied to explore functional groups (e.g., alcohols, amines, carboxylic acids, and ketones) and to examine chemical structures of coal mine dust in the workplace. It is nondestructive and usually applied to membrane filters. FTIR spectrometry identifies molecular vibrations and the resulting absorption by illuminating the sample with multiple-wavelength radiation from an IR-emitting source [64,136]. The concentration is proportional to the absorbance as determined by Beer’s law [137]. The IR is absorbed when incident radiation is equivalent to the energy of a particular molecular vibration. The transmitted radiation is detected and processed based on a “Fourier-transform algorithm” to generate absorbance spectra. Similarly to other spectroscopic techniques, FTIR absorbance can be estimated by: (1) peak height after subtraction of estimated filter background spectra; (2) peak height after spectral blank subtraction; and (3) integrated absorbance after blank corrections [55].

Both MSHA [53] P-7 and NIOSH [54] 7603 methods use a low-temperature radio-frequency asher to destroy the organic matrix of the filter sample, but kaolinite remains structurally unaltered and can interfere with the silica measurement [138]. MSHA method P-7 integrates the absorbance peak area with a baseline between 770 and 815 cm^−1^ for quartz (plus kaolinite) and between 900 and 930 cm^−1^ for kaolinite, whereas NIOSH method 7603 uses a peak height at 800 cm^−1^ with baseline between 670 and 820 cm^−1^ for quartz (plus kaolinite) and a peak height at 915 cm^−1^ with baseline between 860 and 960 cm^−1^ for kaolinite. The absorption peak centered around 800 cm^−1^ is corrected for the kaolinite interference using the absorption centered around 915 cm^−1^. For samples containing >20% of calcite or graphite, a muffle furnace heated at 600 to 800 °C can also be used in NIOSH method 7603. Ashing at these high temperatures allows the conversion of kaolinite to amorphous meta-kaolin that does not interfere with the RCS measurement [138,139]; the kaolinite correction is not needed under this condition. A consistent approach to integrate FTIR absorption peaks is needed to determine deviations among these methods.

FTIR absorption bands for α-quartz at 780 and 799 cm^−1^ shown in Table S3 are similar to the 778 and 797 cm^−1^ identified by Abbasi et al. [131]. Secondary absorbance of cristobalite at 796 cm^−1^ may overlap with wave numbers of 798 and 799 cm^−1^ for quartz. However, minerals that interfere with RCS analysis are typically not found in RCMD, with the exception of kaolinite [138].

Table 4 summarizes infrared absorbance spectra for five membrane filter types [140]. The lowest absorbance was 0.057 at 695 cm^−1^ for polycarbonate filters. For PTFE, absorbances were 0.074 at 779 cm^−1^ and 0.063 at 798 cm^−1^. DM-450 filter absorbances were 0.078 at 779 cm^−1^ and 0.074 at 798 cm^−1^. Lorberau [141] reported that PVC-copolymer filters (DM-450 and DM-800, Gelman Sciences, Ann Arbor, MI, USA) had the lowest absorbance (1.3%–2.4%) and lowest associated standard deviations at wave numbers of 779 cm^−1^ and 798 cm^−1^ for fifteen 25 mm filters that were tested. The DM-450 PVC-copolymer filters have been used for redepositing in both the MSHA P-7 and NIOSH 7603 methods. However, this type of filter is no longer commercially available, and alternative filters are being sought [52]. Absorption for four types of blank membrane filters (i.e., polypropylene, nylon, DM-450, and PVC) from 12 replicate FTIR measurements by Farcas et al. [52] show show smooth absorbances with the least sample-to-sample variations for DM-450 filters while PVC filters show the largest variations among measurements. Multiple absorption peaks are found for both polypropylene and nylon blank filters.

**Figure 5.**
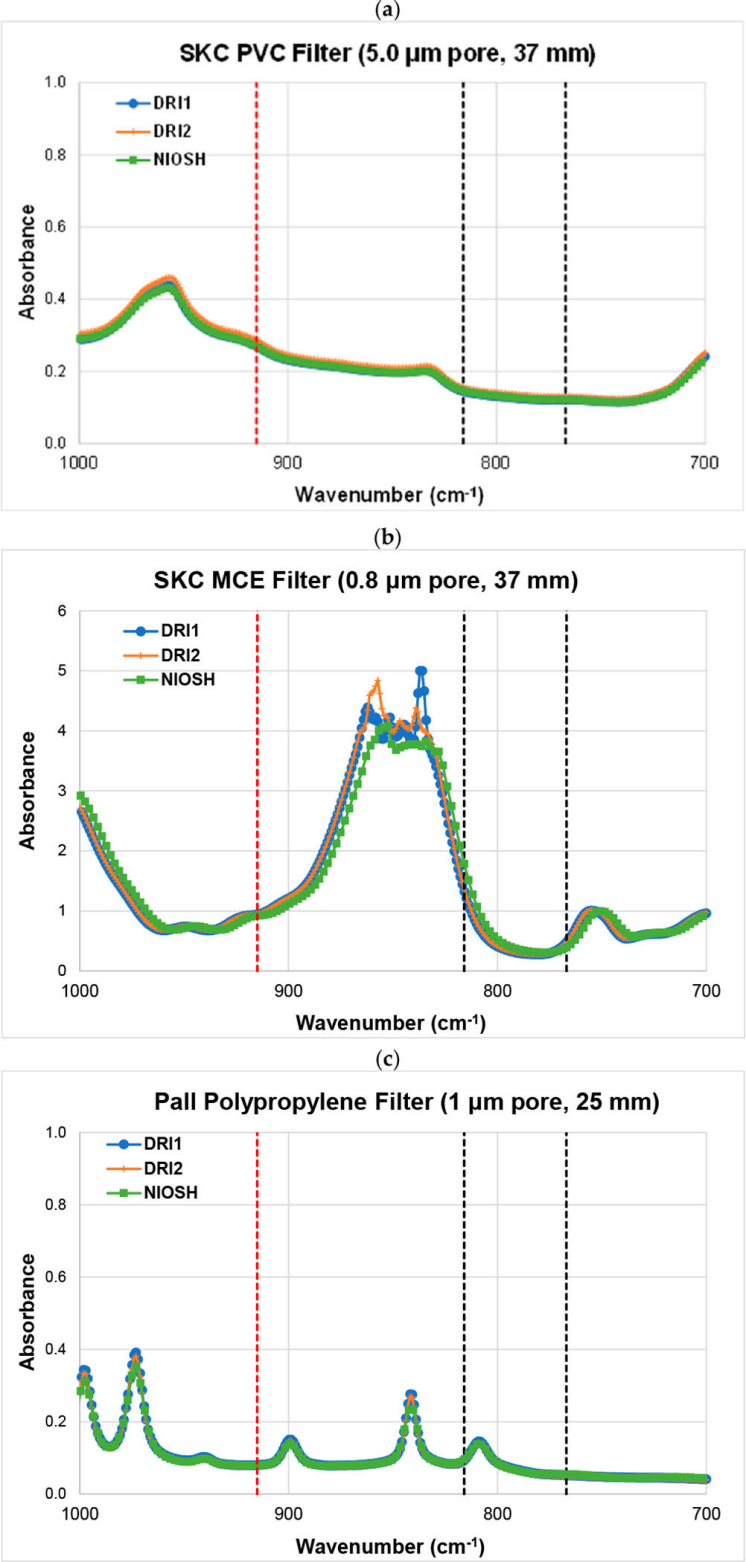
Comparison of blank membrane filters between the NIOSH (Spokane, WA, USA) and DRI (Reno, NV, USA) laboratories for: (**a**) PVC, (**b**) MCE, and (**c**) polypropylene filters. The two vertical black dashed lines bracket RCS absorption from 767 to 816 cm^−1^. The vertical red dashed line represents the wave number (915 cm^−1^) for correcting kaolinite absorption interference for RCS determination.

Interlaboratory comparisons between NIOSH (Spokane, WA, USA) and DRI (Reno, NV, USA) laboratories (Figure 5) also show multiple peaks for polypropylene filters. Nevertheless, good reproducibility in the peaks and valleys of the FTIR spectra was found between the two laboratories. The SKC 5 μm PVC filters (Figure 5a) show the lowest and most stable baseline for RCS determination. The spectrum for SKC 0.8 μm MCE blank filters in Figure 5b shows a sloping tail between the two black vertical lines and a large peak close to 850 cm^−1^ that might interfere with RCS quantification.

Figure S8 shows that the Teflon-coated glass-fiber filter (TX40HI20) used in PDM3700 has high background (~2 absorbance units) in the RCS range (767–816 cm^−1^). Griffiths and de Haseth [136] recommend <0.7 absorbance units for FTIR. Therefore, the TX40HI20 may not be suitable for RCS quantification by FTIR. Additional tests are needed to verify the relative differences between the RCS signal magnitude and blank filter absorbance.

#### 4.1.2. Raman Spectroscopy

Raman spectroscopy uses scattered light to determine the crystallinity and molecular interaction between light and chemical bonds by a vibrational spectrum. It is sensitive to both chemical and morphological changes in samples and capable of selectively identifying specific vibrational modes in organic and inorganic substances between polymorphs. Raman spectroscopy can effectively differentiate between polymorphs and microcrystalline silica [142]. It has been used to determine trace impurities in mineral samples with sharp bands for crystalline minerals and broad bands for amorphous phases or fluorescence [16,19]. Since the spatial resolution of FTIR spectroscopy is limited to 10–20 μm [143], Raman spectrometry complements FTIR analysis by providing better resolution down to 1 μm with micrometer-scale characterization, thereby improving the detection limit.

Stacey et al. [144] reported a quartz peak at 464 cm^−1^ and a cristobalite peak at 410 cm^−1^ when the sample was excited with a near-infrared laser (785 nm). For coal dust analysis, the two major bands are in the regions of 1355–1380 cm^−1^ (D-band) and 1557–1620 cm^−1^ (G [graphic] band) [145]. Shin and Chung [146] advocate the application of wide-area illumination to enhance reproducibility and the signal-to-noise ratio of spectra for optimal Raman spectroscopic analysis.

**Figure 6.**
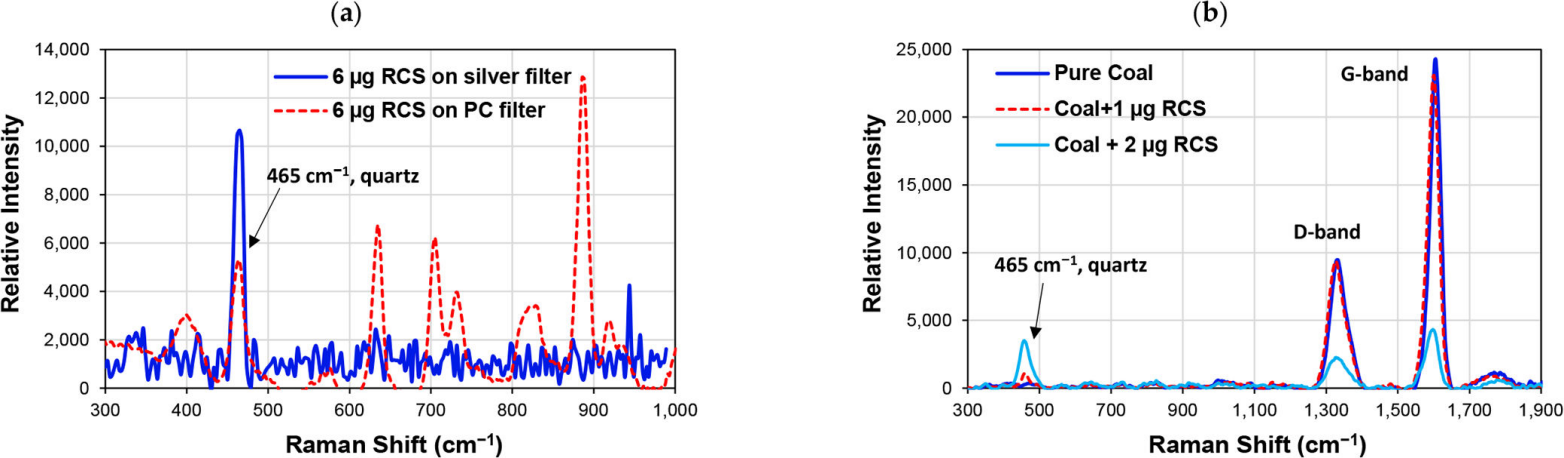
Raman spectra for: (**a**) crystalline silica on silver-membrane and polycarbonate (PC)-membrane filters; and (**b**) samples of pure coal and coal mixed with 1 μg and 2 μg of RCS on a PC filter.

Preliminary laboratory tests of polycarbonate and silver membrane filters for RCS were conducted using an iRaman Plus spectrometer (B&W Tek) with 532 nm laser excitation. Figure 6a shows that polycarbonate and silver membrane filters do not cause spectral interference with the quartz peak at 465 cm^−1^. The quartz peak intensity on the silver filter is twice that for the polycarbonate filter with a given mass loading (6 μg RCS), which is attributed to the enhanced Raman scattering from porous silver membrane filters [147]. Figure 6b shows that the iRaman Plus (532 nm) is capable of detecting quartz (characteristic band at 465 cm^−1^) and black coal (characteristic bands at 1356 cm^−1^ and 1576 cm^−1^) when a small amount of quartz is added to coal and that the black coal carbon does not interfere with quartz. However, a separate test with a low-ranked brown coal sample with rich volatile organics generated high fluorescence signals that saturated the detector. As the excitation laser wavelength of a Raman spectrometer affects excitation efficiency, Raman scattering intensity, fluorescence, and heating, further research is needed to select the best laser wavelength for RCS quantification in coal matrix. Limited research has been done with Raman spectroscopy [142,148–150] for coal mines, and more tests are needed on membrane filters to better understand the blank filter absorbance and potential spectral interference.

### 4.2. Detection Limits

Filters should have low blank levels for the targeted chemical species (<1 μg per 47 mm filter). Background contents of major and trace elements and isotopic composition for the PTFE, nylon, polycarbonate, and glass-fiber filters are reported by Lee et al. [151]. Trace-element concentrations are the lowest in the PTFE followed by nylon.

Prior to sampling, at least 2% of each batch of 100 filters from each manufacturer should be light-inspected for pinholes, creases, discoloration, or other defects and analyzed for all species to verify the background concentrations. Quartz-fiber filters adsorb organic vapors requiring prefiring at 900 °C for 4 h prior to “acceptance testing.” Nylon and MCE membrane filters absorb HNO_3_ over time that need to be acceptance tested and/or washed with distilled deionized water prior to sampling [96].

The nominal RCS limits of detection (LODs) are 4 and 10 μg per 37 mm PVC sample for MSHA P-7 and NIOSH 7603 methods, respectively [53,54]. Linear calibration curves between 10 and 1000 μg for FTIR can be obtained with standard reference materials (SRM 1878a for quartz and SRM 1879a for cristobalite [152]). Lorberau [141] reported LOD levels (triple the standard deviation of the average blanks) for DM-450 and DM-800 of 5.3 μg at 779 cm^−1^ and 2.9 μg at 798 cm^−1^ per 25 mm filter with 1.3%–2.4% variations within and between the two filter types. These levels are higher than the 0.59–0.99 μg per 47 mm filter in Table 5, reported by Farcas et al. [52]. This may be due to the fact that filters are prewashed in isopropyl alcohol prior to FTIR analysis [52]. Laboratory tests should be conducted to determine if rinsing or prewashing in isopropyl alcohol is needed prior to field sampling.

Both Zefon 5 μm PVC and Sterlitech 0.45 μm polypropylene reported lower LODs (0.52–0.72 μg per 47 mm filter) by MSHA [53] P-7 than by NIOSH [54] 7603 (1.2–1.5 μg/filter). Note that LODs varied by around threefold for 5 μm PVC filters between the MSHA P-7 and NIOSH 7603 methods. However, the trend is reversed for DM-450 and Nylon filters, with NIOSH 7603 having lower LODs than MSHA P-7. The limit of quantification (LOQ, 10 times the standard deviation of the average blanks) in Table 5 are lower than LODs of ~7 μg/filter for quartz and 20 μg/filter for coal dust reported in Tuchman et al. [29] and 3–10 μg/filter for quartz reported by Abbasi et al. [131] and NIOSH [153]. The LOD should account for filter variations by batches as well as measurement variations due to changes in environmental parameters and FTIR performances.

The LOD by Raman spectrometry can be improved with longer collection times for several locations on each filter. In general, detection limits for Raman spectroscopy can be one to two orders of magnitude lower than those for XRD and FTIR. For five 13 mm PVC (5 μm pore size) filters, Stacey et al. [144] reported LOD of 0.049 μg/filter for quartz and 0.02 μg/filter after accounting for the variability of the background scatter. For an analysis area of 100 μm^2^, Stacey et al. [144] reported an LOQ of 0.066–0.161 μg/filter for quartz by analyzing 50 locations on 5 mm-diameter silver-membrane-filter particle deposits. The measurement precision was 10%–25% for 0.25–10 μg quartz. Using an aerosol microconcentrator to obtain 400 to 1000 μm diameter deposits on silver membrane filters, Zheng et al. [147] reported 8 to 55 ng α-quartz LODs using a portable Raman spectrometer at 465 cm^−1^ over 60 s integration times.

## 5. Major Findings and Recommendations

Mine safety regulations require monitoring dust and RCS concentrations in mining environments. CMDPSUs are used to collect dust samples for gravimetric and RCS quantification in offsite laboratories. However, it typically takes days or weeks before the data are available. The PDM measures dust concentrations in near real time. However, the current filter used in the PDM and the filter holder design are not suitable for RCS measurement. This study reviews characteristics of different filter media with an aim to identify filters that can be used for both dust mass concentration and chemical composition analysis, especially for RCS quantification by FTIR and Raman spectrometry.

Chemical and physical characteristics of 12 types of commercially available filter substrates are evaluated (Table S1). Among the three main classes of filters that have been commonly applied for aerosol sampling, the five fibrous filters, including the Teflon-coated glass fiber (TX40HI20) used in the PDM3700, are not further considered for mass and quartz determination owing to their low and variable collection efficiency (Table 2) and/or interference with quartz measurement. Adsorption or desorption of SO_2_ and NO_2_ on glass fibers [120–122] and adsorption and volatilization of organic vapors in quartz-fiber filters [96] may bias mass determination. Quartz-fiber filters have been applied to long-term sampling in PM_2.5_ speciation networks, but they contain quartz material in the filter mat and cannot be used for RCS quantification.

Performance of capillary pore filters (i.e., polycarbonate) shows variable collection efficiency (as low as ~1%–6% for 5 μm and 8 μm pore sizes) [22]; high penetration (0.4 and 8 μm pore sizes) for particles between 10 and 100 nm [106,107]; and variable blank filter absorbance by FTIR spectrometry [52].

Different test results are reported for the six porous membrane filters. Silver membrane has variable collection efficiency (~25%–90%) and high costs per filter. For 5 μm silver membrane filters, as low as 25% collection efficiency was reported by Liu et al. [22] and as low as 42% efficiency was found by Soo et al. [107]. Pressure drops and filtration efficiency increase with decreasing pore size. Silver membrane filters are not transparent by FTIR and are not suitable for FTIR measurements using the transmission mode; further testing is needed to verify if they can be used in reflectance mode. The ability of these filters to enhance Raman scattering makes them good candidates for Raman measurement [147].

MCE membrane filters show the highest collection efficiency (98%–99.99%) [107] and low penetration (~0.1%) [106]. However, MCE filters absorb nitric acid. Keck and Wittmaack [121,122] reported adequate sampling of ammonium salts with MCE filters, as it retained a large fraction of volatilized gases. Comparing gravimetric mass among Teflon, PVC, MCE membrane filters and glass-fiber filters, Tsai et al. [126] reported the largest coefficient of variation (CV, 0.27%) for MCE (Table S2), one to two orders of magnitude higher than those of Teflon-membrane and PVC filters, an indication of potential water uptake by MCE filters. Therefore, the chemical stability of MCE filters warrants further examination. Preliminary laboratory tests show a sloping tail close to 850 cm^−1^ that might interfere with RCS quantification by FTIR. MCE filters are made of cellulose nitrate and small amounts of cellulose acetate. It is not clear if consistent mixtures are used among the five different vendors. For example, Table S1 shows that the Thermo Scientific Nalgene MCE contains a mixture of cellulose diacetate and triacetate. More tests need to be conducted to select an MCE vendor and to ensure blank filter consistency among batches.

No collection efficiency was reported for nylon filters. Nylon filters are known to absorb nitric acid. Farcas et al. [52] reported higher RCS limit of detection (1.5–1.8 μg per 47 mm filter) in 0.45 μm nylon filters than those of 0.45 μm DM-450 or polypropylene, and 5 μm PVC filters. Blank nylon filters show multiple peaks over the RCS analysis region on FTIR. Inhomogeneous particle deposition was reported when redeposit of ashed nylon filters [52].

Little testing has been conducted on polypropylene filters. In laboratory tests of blank filters, multiple absorption peaks with large variations were found on polypropylene filters by Farcas et al. [52] as well as at NIOSH (Spokane, WA, USA) and DRI (Reno, NV, USA) laboratories that might interfere with RCS quantification.

Collection efficiency for 5 μm PVC membrane filters used in the CMDPSU are generally high, ranging 92.98%–99.95% on individual tests among filters from three vendors (i.e., SKC, Gelman, and Millipore), with the lowest (92.98%) found at 20.5 cm/s face velocity for SKC filters [107]. Very low collection efficiency (as low as 49%) was also found for the 5 μm Metricel PVC filter at a higher face velocity (>51 cm/s) by Liu et al. [22]. Collection efficiency of 5 μm PVC for PDM3700 at a high face velocity of 27.6 cm/s needs to be tested for mass and RCS determination by PDM. Farcas et al. [52] cautioned that 5 μm PVC filters may replace the DM-450 filter for coal dust quartz analysis by FTIR, but their ability to retain small particles warrants additional experiments.

Large variations in FTIR baseline absorption of blank PVC filters were reported by Farcas et al. [52]. However, preliminary laboratory testing at both NIOSH (Spokane, WA, USA) and DRI (Reno, NV, USA) laboratories showed stable and low baselines for the RCS spectrum of 767–816 cm^−1^. Low detection limits of 0.52–1.5 μg per 47 mm 5 μm Zefon PVC filters were reported by Farcas et al. [52], lower than the LOD of ~7 μg/filter for quartz noted by Tuchman et al. [29] and 3–10 μg/filter for quartz reported in Abbasi et al. [131] and NIOSH [153].

Overall performance is best for PTFE Teflon-membrane filters, with an average of 99% ± 2% collection efficiency for the 171 tests [107]. The lowest collection efficiency of 94.76% at 3.11 cm/s was found for 5 μm Pall PTFE filters. Although earlier tests by Liu et al. [22] reported >88% collection efficiency for the 3 μm Zefluor filters and >85% for 5 μm Gelman filters, the 2 μm PTFE produced adequate collection efficiency with >94.6% (Zefluor) and >99.89% (Ghia). Lorberau [140] reported low IR absorbance for PTFE Teflo by FTIR (0.074 to 0.078 at 779 cm^−1^ and 0.063 to 0.074 at 798 cm^−1^), at similar magnitude to the 0.074–0.078 level found in DM-450 (a PVC-acrylic copolymer).

Two candidate filters, PTFE and PVC, warrant additional tests. Potential vendors should be contacted to confirm the availability and cost of 13 mm diameter filters to ensure long-term consistency and stability. Currently, Pall Corporation supplies 13 mm diameters of 0.45 μm and SKC supplies 5 μm PTFE membrane filters. Given the high face velocity in PDM3700, the 2 μm Teflo™ from Pall Corporation with thickness of 46 μm, most commonly applied for aerosol sampling in U.S. networks, may be considered. The challenge is to manufacture a 13 mm PTFE filter with thinner polymethylpentene (PMP) support ring to prevent leaks. It may be possible to stack two PTFE filters without a support ring. The thickness of 2 μm Zefluor (152 μm) is three times that of Teflo, which may not transmit light for FTIR analysis.

Filter media testing and selection should consider the characteristics required for mass, FTIR, and Raman measurements. For mass determination by the PDM, the filters need to have high filtration efficiency (≥99% for the most penetrable particle sizes) and a reasonable pressure drop (up to 16.7 kPa) to accommodate high dust loadings [29]. Additional requirements include: negligible uptake of water vapor and gaseous volatile compounds; adequate particle adhesion as a function of particle loading; sufficient particle loading capacity to form a stable particle deposit layer during sampling in wet and dusty environments; mechanical strength to withstand vibrations and pressure drops across the filter; and appropriate filter mass compatible with the tapered element. FTIR and Raman measurements require filters to be free of spectral interference. Furthermore, because the irradiated area does not completely cover the sample deposit, particles should be uniformly deposited on the filter.

For a broader application of potential chemical characterization, two out of 100 filters should be submitted for light inspection and acceptance testing of trace elements and ions. Standard operating procedures for FTIR and Raman spectroscopy need to be developed. Detection limits and blank filter absorption and potential interference by FTIR and Raman spectrometry need to be determined.

## Supplementary Material

Supplementary

## Figures and Tables

**Table 1.** Specification of MSHA coal mine dust monitors [39].

Sampler Type	Coal Mine Dust Personal Sampling Units (CMDPSU)	Personal Dust Monitor (PDM 3700)
**Manufacturer**	Zefon International, Ocala, FL, USA (zefon.com, accessed on 1 October 2022)	Thermo Fisher, Waltham, MA, USA (thermofisher.com, accessed on 1 October 2022)
**Averaging Time**	Integrated 8 h sample	Real time with 30 min average
**Size-selective Inlet (d_50_ of 4 μm)**	Dorr-Oliver nylon cyclone by Zefon	Higgins Dewell (HD) cyclone (Model BG14CP conductive plastic by Mesa Labs, Lakewood, CO, USA, mesalabs.com accessed on 1 October 2022)
**Filter Cassette Assembly**	2, 3, or 4 piece conductive housing cassettes	14 mm circular polypropylene base with a hollow axial stem
**Flow Rate**	2.0 L/min (±5% in mine) for coal	2.2 L/min (±2.5%)
**Operating Temperature**	0 to 45 °C	−20 to +40 °C
**Filter Type (Diameter)**	37 mm, 5 μm pore size polyvinyl chloride (PVC)	13 mm Teflon-coated borosilicate glass-fiber filter (TX40HI20, Pall Corporation, East Hills, NY, USA, pall.com, accessed on 1 October 2022)
**Filter Exposed Area**	784 mm^2^	132.7 mm^2^
**Face Velocity**	4.25 cm/s	27.6 cm/s
**Mass Loading Range**	0–200 mg	0.1–4 mg
**Mass Determination Method**	Gravimetry	Real-time TEOM inertial microbalance
**Accuracy**	Accurate measurements are possible within ±5%	±25% of the reference method for concentrations > 0.2 mg/m^3^
**Physical Dimensions**	Sampling Tube: 92 cm Cyclone Assembly: 6 cm (d) × 15 cm (h) × 6 cm (w) Pump: 5.7 cm (d) × 10.8 cm (h) × 10.2 cm (w)	Sampling Tube: 92 cm Cyclone Assembly: 5.08 cm (w) × 4.32 cm (d) × 9.91 cm (h) Monitor: 24.31 cm (w) × 8.26 cm (d) × 17.15 cm (h)
**Sampler Weight**	Pump: 0.65 kg with battery pack	2 kg
**Pump Type**	Escort ELF Pump	Internal sampling pump
**Power Requirements**	48 volt battery pack of 4 NiMH cells	Lithium ion battery assembly

**Table 2.** Filter types, pore sizes, permeabilities, and collection efficiency for filters applicable to mine exposure monitors [22].

Filter Type	Filter Material (Manufacturer)	Pore Size (μm)	Filter Permeability Face Velocity ^a^ (cm/s)	Range of Collection Efficiency ^b^

Teflon membrane	Fluoropore (PTFE-polyethylene reinforced, Millipore Sigma, Burlington, MA, USA)	3	23.5	98.2%–99.8%

	Teflon (Gelman Sciences, Hilliard, OH, USA)	5	56.8	85%–99.9%

	Teflon (Ghia SKC, Eighty Four, PA, USA)	2	23.4	99.89%–99.99%
		3	24.2	92%–98.98%

	Teflon (Zefluor, Millipore Sigma, Burlington, MA, USA)	2	32.5	94.6%–99.96%
		3	31.6	88%–99.9%

Silver membrane	Pure metallic silver (Sterlitech, Auburn, WA, USA)	0.45	1.8	93.6%–99.98%
		0.8	6.2	90%–99.6%
		1.2	9.2	73%–99.7%
		5	19.0	25%–99.2%

Polyvinyl chloride (PVC) membrane	PVC (Metricel)	0.8	2.7	99.96%–>99.99%
	PVC (Metricel)	5	51	49%–98.8%
	PVC (Millipore Sigma, Burlington, MA, USA)	2	5	88%–99.9%
	PVC-5 (Millipore Sigma, Burlington, MA, USA)	5	11	96.7%–>99.9%

Cellulose acetate/nitrate membrane	MF-RA (Millipore Sigma, Burlington, MA, USA)	1.2	6.2	>99.9%
	MF-SS (Millipore Sigma, Burlington, MA, USA)	3	7.5	98.5%–99.9%
	MF-SM (V)	5	10	98.1%–99.9%
	MF-SC (Millipore Sigma, Burlington, MA, USA)	8	14.1	92%–99.9%

Capillary pore membrane	Polycarbonate (Nuclepore, Whatman-Cytiva, Little Chalfont Buckinghamshire, UK)	0.4	2.9	78%–99.99%
		0.6	2.1	53%–99.5%
		5	30.7	6%–90.7%
		8	21.2	1%–90.5%

Quartz fiber	2500 QAO (Pallflex, Pall Corp., Duncan, SC, USA)	NA	41	84%–99.9%

Teflon-coated glass fiber	TX40HI20 (Pall Corp., Duncan, SC, USA)	NA	15.1	92.6%–99.6%
	TX40HI20 (2nd lot, Pall Corp., Duncan, SC, USA)	NA	9	98.9%–99.9%

Cellulose fiber	Whatman 40 (Whatman-Cytiva, Little Chalfont Buckinghamshire, UK)	NA	3.7	77%–99.99%
	Whatman 41 (Whatman-Cytiva, Little Chalfont Buckinghamshire, UK)	NA	16.9	43%–99.5%

Glass fiber	Microquartz (Gelman Sciences, Hilliard, OH, USA)	NA	14.1	98.5%–99.99%
	GF/A (Whatman-Cytiva, Little Chalfont Buckinghamshire, UK)	NA	14.5	99%–99.99%

a Filter permeability is characterized by the face velocity measured at a pressure drop of 1.3 kPa (1 cm Hg) across the filter.

b The efficiency range corresponds to particle diameters in the range of 0.035–1 μm, a pressure drop of 1.3–40 kPa (1–30 cm Hg), and a face velocity range of 1–100 cm/s.

**Table 3.** Summary of collection efficiency on membrane and capillary pore filters over a range of pore sizes and flow rates [107].

	Filter Type ^a^				
	PTFE	PVC	Silver Membrane	Polycarbonate	MCE

No. of filters tested	171	171	168	171	162
Median	99.86%	99.74%	96.07%	98.01%	99.99%
Mean ± standard deviation	99.02 ± 2.25%	98.85 ± 2.96%	86.46 ± 20.3%	85.32 ± 22.2%	99.5 ± 4.76%
Lowest collection efficiency	94.76%	92.98%	42.10%	22.48%	98.82%
Pore size ^b^	5 μm	5 μm	5 μm	5 μm	0.45 μm
Flow rate ^b^	1.7 L/min	11.2 L/min	4.4 L/min	1.7 L/min	2.5 L/min
Face velocity ^b,c^	3.11 cm/s	20.5 cm/s	8.06 cm/s	3.11 cm/s	4.58 cm/s
Vendor ^b^	Pall	SKC	Sterlitech	Millipore	SKC

a PTFE: polytetrafluoroethylene (0.45, 1.2, and 5 μm); PVC: polyvinyl chloride (0.8 and 5 μm); MCE: mixed cellulose esters (0.45, 0.8, 1.2, and 5 μm); polycarbonate (0.4, 0.8, 2, and 5 μm); and silver membrane (0.45, 0.8, 1.2, and 5 μm).

b Conditions corresponding to the lowest collection efficiency.

c Based on exposure area of 9.1 cm^2^ in the 37 mm cassette.

**Table 4.** Infrared absorbance of five 25 mm-diameter membrane filters [140].

			Average Absorbance ^b^ at Selected Quartz Bands ^c^		
Filter Name	Filter Type ^a^	Gravimetric Weight	695 cm^−1^	779 cm^−1^	798 cm^−1^
Nuclepore	PE	4.90 mg	0.057 ± 0.005	0.116 ± 0.004	0.157 ± 0.003
Teflo	PTFE	4.50 mg	0.157 ± 0.021	0.074 ± 0.011	0.063 ± 0.010
DM-450 (0.45 μm pore size)	PVC/A	12.60 mg	0.163 ± 0.003	0.078 ± 0.001	0.074 ± 0.001
DM-800 (0.8 μm pore size)	PVC/A	14.13 mg	0.205 ± 0.005	0.111 ± 0.003	0.108 ± 0.003
GLA-5000	PVC/A	5.31 mg	0.222 ± 0.005	0.105 ± 0.006	0.112 ± 0.006

a PTFE = polytetrafluoroethylene; PVC = polyvinylchloride; PVC/A = polyvinylchloride–acrylonitrile copolymer; and PE = Nuclepore polyester.

b FTIR identify functional groups by measuring absorption of infrared radiation as a function of wavelengths. Absorbance (A) is a unitless measure of the optical density: A = log_10_(I_0_/I), where I_0_ is the intensity of incident light and *I* is the intensity of transmitted light. Absorbance is defined by Beer’s law and is linearly proportional to the concentration of light-absorbing species.

c Analyzed by Nicolet 60-SX FTIR spectrometer.

**Table 5.** Limits of detection (LOD) and limits of quantification (LOQ) for membrane filters analyzed by FTIR [52].

	LOD (μg) ^a^		LOQ (μg) ^a^	

47 mm Filter Type ^b^ (Pore Size)	MSHA ^c^	NIOSH ^c^	MSHA ^c^	NIOSH ^c^

DM-450 (0.45 μm)	0.99	0.59	3.3	2
PVC (5 μm)	0.52	1.5	1.7	4.9
Polypropylene (0.45 μm)	0.72	1.2	2.4	4
Nylon (0.45 μm)	1.8	1.5	6	4.9

a Standard deviations of 12 blank filters in 47 mm diameter (after rinsing with isopropyl alcohol) determined by FTIR at 800 cm^−1^ absorbance (LOD = 3 times standard deviations of the average blanks, LOQ = 10 times standard deviations of the average blanks) [52].

b DM-450: a vinyl/acrylic co-polymer membrane filter from Pall Life Science; polyvinylchloride (PVC 547) from Zefon; polypropylene (PP04547100) from Sterlitech; and Nylon (HNWP04700) from Millipore.

c MSHA Method P-7 [53]; NIOSH Method 7603 [54].

## Data Availability

Relevant data are contained in the cited articles and reports.
