# Supplementary material for: Review of Filters for Air Sampling and Chemical Analysis in Mining Workplaces
Source: Minerals (Basel). Author manuscript; Available in PMC 2023 May 11. (PMC10174218; doi:10.3390/min12101314)
Supplement: Supplementary [file NIHMS1846801-supplement-Supplementary.pdf]

Review

Supplemental Materials: Review of Filters for Air Sampling and Chemical Analysis in Mining Workplaces

Judith C. Chow *, John G. Watson, Xiaoliang Wang, Behrooz Abbasi, Wm. Randolph Reed and David Parks

S1. Supplemental Tables

Table S1. Summary of filter characteristics for sampling and analysis.

Table S2. Mass concentration under uncontrolled and controlled temperature and humidity environments.

Table S3. Major infrared bands of the principal polymorphs in crystalline silica.

Table S1. Summary of filter characteristics for sampling and analysis.

Filter Type	Filter Characteristics and Analysis Methods	Trade Name,<br>Supplier,<br>Website, and<br>Quantity Available	Filter Diameter	Pore Size (Thickness)	Cost US ($)	Comments
		i) Porous Membrane Filters				
PTFE (Polytetrafluoroethylene)	**Physical Characteristics**<br>• Thin film microporous membrane with synthetic fluoropolymer or polytetrafluoroethylene stretched between polymethylpentene (PMP) support ring.<br>• Thin membrane with nearly transparent white surface.<br>• Minimal dispersion of transmitted radiation.<br>• High particle collection efficiencies.<br>• High mechanical strength.<br>• Low particle loading capacity.<br>• Limited temperature range. Melts at ~60 °C.<br>• High flow resistance and pressure drop.<br><br>**Chemical Characteristics**<br>• Usually low blank levels.<br>• Composed of carbon-based material; inappropriate for carbon analysis.	Measurement Technology Laboratory (MTL), Minneapolis, MN, USA<br>mtlcorp.com, accessed on 1 October, 2022<br>PT47 polytetrafluoroethylene<br>with fluorinated ethylene propylene (FEP) support ring<br>Package of 50	46.2 mm	2 µm	H	• Complies with U.S. 40CFR1065 requirement for PTFE filters used in U.S. Federal Reference Method (FRM) network for $PM_{2.5}$ (particles with aerodynamic diameter less than 2.5 µm) sampling and mass determination.
		Omnipore™<br>Millipore Sigma, Burlington, MA, USA<br>sigmaaldrich.com<br>Package of 100	47 mm	0.2 and 1.5 µm	M	
		Omnipore™<br>Millipore Sigma<br>fishersci.com accessed on 1 October, 2022	47 mm	0.1, 1, 5, and 10 µm	M	
		Teflo™<br>Pall Corporation, East Hills, NY, USA<br>us.VWR.com accessed on 1 October, 2022<br>Package of 25, 50, and 100	25 mm, 37 mm, and 47 mm	1 µm (76 µm), 2 µm (46 µm), and 3 µm (30.4 µm)	H	• Most commonly applied for aerosol sampling in U.S. networks.

**Filter Type**	**Filter Characteristics and Analysis Methods**	**Trade Name, Supplier, Website, and Quantity Available**	**Filter Diameter**	**Pore Size (Thickness)**	**Cost US ($)**	**Comments**
	• Inert to gas adsorption with volatilization of nitrite and nitrate.<br>• Low hygroscopicity.<br>• Low blank weight.<br><br>**Compatible Analysis Methods**<br>• Gravimetry, OA, XRF, PIXE, INAA, AAS, ICP/AES, ICP/MS, IC, AC, FTIR, and Raman	TF (PTFE)<br>Pall Corporation<br>us.VWR.com, accessed on 1 October, 2011<br>Package of 25, 50, and 100	13 mm<br><br>25 mm and 47 mm<br><br>142 mm<br><br>293 mm	0.45 µm<br><br>0.2, 0.45, and 1 µm<br><br>0.2 and 0.45 µm<br><br>0.2 µm	M (up to 25 mm), H (37 mm and over)	• For air and gas filtration and ventilation application.<br>• Suitable for strong acids and solvents.
		Zefluor™<br>Pall Corporation<br>pall.com, accessed on 1 October, 2022<br>Package of 25, 50, and 100	25 and 47 mm<br><br>25, 37, 47, and 90 mm diameter, and 20.3 x 25.4 cm^2<br><br>50 and 90 mm diameter, and 20.3 x 25.4 cm^2<br><br>37 (w/ support pad) and 47 mm	0.5 µm (178 µm)<br><br>1 µm (165 µm) and 2 µm (152 µm)<br><br>3 µm (152 µm)<br><br>5 µm	NA	• Thick PTFE membrane filter, discontinued at VWR, Sigma Aldrich in U.S.

Filter Type	Filter Characteristics and Analysis Methods	Trade Name, Supplier, Website, and Quantity Available	Filter Diameter	Pore Size (Thickness)	Cost US ($)	Comments
		SKC PTFE, Eighty Four, PA, SA<br>skcinc.com, accessed on 1 October, 2022<br>Package of 50, 100, 150, or 250	13 mm<br>25 mm<br>37 mm<br>47 mm	5 µm<br>0.5, 1, 2, and 3 µm (w/ PMP support ring)<br>0.3, 0.45 (w/ polypropylene support), 1, 2 (w/ PMP support ring), 5, and 10 µm (w/ polypropylene support ring)<br>0.5, 2 (w/ PMP support ring), and 5 µm	L to M	
		Whatman PTFE, Florham Park, NJ, USA<br>sigmaaldrich.com, accessed on 1 October, 2022<br>Package of 50	47 mm	0.2 µm	H	• Suitable for temperatures up to 120 °C.
PP (Polypropylene)	**Physical Characteristics**<br>• Thermoplastic polymer made of monomer propylene<br>• Thin membrane with	Metricel™<br>Pall Corporation<br>us.VWR.com, accessed on 1 October, 2022<br>Package of 100	25 mm	0.1 µm	L	

Filter Type	Filter Characteristics and Analysis Methods	Trade Name, Supplier, Website, and Quantity Available	Filter Diameter	Pore Size (Thickness)	Cost US ($)	Comments
	nearly transparent white surface.<br>• High temperature resistance (82 °C).<br><br>**Chemical Characteristics**<br>• Inert to gas adsorption.<br>• Low hygroscopicity.<br>• Low extractables.<br><br>**Compatible Analysis Methods**<br>• Gravimetry, OA, XRF, PIXE, INAA, AAS, ICP/AES, ICP/MS, IC, AC, FTIR, and Raman	Zefon International, Ocala, Fl, USA<br>zefon.com, accessed on 1 October, e0ww<br>Package of 50	37 mm	NA	L	
PVC (Polyvinyl Chloride)	**Physical Characteristics**<br>• Thin membrane of PVC polymer.<br>• White opaque surface, diffuses transmitted light.<br>• Melts at ~100 °C.<br>• Low flow resistance.<br>• Potential electrostatic buildup.<br><br>**Chemical Characteristics**	Millipore<br>emdmillipore.com, accessed on 1 October, 2022<br>Package of 100	47 mm	5 µm (50 µm)	H	• Specified by ASTM, NIOSH, and OSHA in monitoring and analyze for silica, quartz, and carbon black.
		GLA-5000<br>Pall Corporation<br>us.VWR.com, accessed on 1 October, 2022<br>Package of 100	25, 37, and 47 mm	5 µm	L to M	• Sampling of metals, silica, and dust.

Filter Type	Filter Characteristics and Analysis Methods	Trade Name, Supplier, Website, and Quantity Available	Filter Diameter	Pore Size (Thickness)	Cost US ($)	Comments
	• Dissolves in some organic solvents.<br>• Low hygroscopicity.<br>• Low blank weight.<br><br>**Compatible Analysis Methods**<br>• XRD, FTIR, and Raman	Zefon<br>zefon.com, accessed on 1 October, 2022<br>Package of 100<br><br>Package of 10	25, 37, and 47 mm diameter, and<br><br>20.3 x 25.4 cm^2	5.0 μm	L	
Nylon Membrane	**Physical Characteristics**<br>• Thin membrane of diacid chlorides, diamines, polyamide, or thermoplastic polymers.<br>• White opaque surface, diffuses transmitted light.<br>• Melts at 60 °C.<br>• High flow resistance.<br><br>**Chemical Characteristics**<br>• High HNO_3 collection efficiency.<br>• Passively adsorbs low levels of NO, NO_2, PAN, and SO_2.<br>• Low hygroscopicity.<br>• Low blank weight.<br><br>**Compatible Analysis Methods**<br>• IC, AC, FTIR, and Raman	Ultipore<br>Pall Corporation<br>us.VWR.com, accessed on 1 October, 2022<br>Package of 100	47 mm	0.45 μm	L	• Reinforced single layer of N66 Nylon with nonwoven internal polyester<br>• Used for collecting particles for ion analysis in the U.S. IMPROVE and CSN networks.
		Restek Corp, Bellefonte, PA, USA<br>us.VWR.com, accessed on 1 October, 2022<br>Package of 100	47 mm	0.22 and 0.45 μm	L	• Reinforced single layer of N66 Nylon with nonwoven internal polyester
		Nalgene<br>Thermo Scientific, Waltham, MA, USA<br>us.VWR.com, accessed on 1 October, 2022<br>Package of 100	47 mm	0.2 and 0.45 μm	L	• Used for aqueous and organic solvent filtration

Filter Type	Filter Characteristics and Analysis Methods	Trade Name, Supplier, Website, and Quantity Available	Filter Diameter	Pore Size (Thickness)	Cost US ($)	Comments
Silver Membrane	**Physical Characteristics**<br>• Thin membrane of sintering, uniform metallic silver particle.<br>• Grayish-white surface with low light transmittance.<br>• Melts at 350 °C.<br>• Conductive with high flow resistance.<br><br>**Chemical Characteristics**<br>• Resistant to chemical attack by all fluids.<br>• Passively adsorbs organic vapors.<br>• Low hygroscopicity.<br>• High blank weight.<br>• Low extractables.<br><br>**Compatible Analysis Methods**<br>• Gravimetry, XRD, FTIR, and Raman	Millipore<br>sigmaaldrich.com, accessed on 1 October, 2022<br>Package of 50	25 mm	0.45 μm	H	• Used for air sampling of carbon black, coal, tar products, coke oven emissions, asbestos, and silica
		SKC<br>skcinc.com, accessed on 1 October, 2022<br>Package of 50	13, 25, 37, and 47 mm	0.2, 0.45, 0.8, 1, 2, 3, and 5 μm (50 μm)	H	• Used for industrial hygiene sampling.<br>• 99.97% pure metallic silver.
		Sterlitech Corporation, Auburn, WA, USA<br>sterlitech.com, accessed on 1 October, 2022<br>Package of 25–100	13, 37, and 47 mm	0.2, 0.45, 0.8, 1.2, 3, and 5 μm	H	
MCE (Mixed Cellulose Esters)	**Physical Characteristics**<br>• Thin membrane of mixed cellulose esters, cellulose nitrate, and cellulose acetate. Some filters include PVC membrane.<br>• White opaque surface diffuses transmitted light.	Advantec Corp, Irvine, CA, USA<br>us.VWR.com, accessed on 1 October, 2022<br>Package of 100	47 or 50 mm	0.2, 0.45, 0.65, 0.8, 1, and 3 μm	L	• For microbiological examination and water sampling, including white, black, and gem membranes with or without hydrophobic edge.

Filter Type	Filter Characteristics and Analysis Methods	Trade Name, Supplier, Website, and Quantity Available	Filter Diameter	Pore Size (Thickness)	Cost US ($)	Comments
	• Melts at ~75–130 °C.<br>• High loading capacity.<br>• Potential electrostatic build up.<br><br>**Chemical Characteristics**<br>• High hygroscopicity.<br>• Negligible ash content.<br>• Dissolves in many organic solvents.<br>• Low chemical resistance.<br>• Low hygroscopicity.<br>• Low blank weight.<br>•Biologically inert.<br><br>**Compatible Analysis Methods**<br>• Gravimetry, OM, TEM, SEM, XRD, FTIR, Raman, and biomedical applications.	Millipore MCE<br>emdmillipore.com, accessed on 1 October, 2022<br>Package of 100	47 mm	1 μm (30–80 μm)	L	
		SKC MCE<br>skcinc.com<br>Package of 100	25 mm	0.8 μm		
		Nalgene MCE<br>Thermo Scientific<br>us.VWR.com, accessed on 1 October, 2022<br>Package of 100	47 mm	0.2 and 0.45 μm	M	• Pharmaceutical industries often use cellulose nitrate filters. Also known as nitrocellulose for molecular biology filtration, prepared by reaction of cellulose and nitric acids.<br>• Cellulose acetate membrane filter is a mixture of cellulose diacetate and triacetate.

Filter Type	Filter Characteristics and Analysis Methods	Trade Name, Supplier, Website, and Quantity Available	Filter Diameter	Pore Size (Thickness)	Cost US ($)	Comments
		Whatman MCE<br>sigmaaldrich.com, accessed on 1 October, 2022<br>Package of 100	25, 47, 100, 110, 142, and 150 mm (ME24)<br><br>25, 47, 50, 90, 100, 110, and 142 mm (ME25)<br><br>25, 37, 47, 50, and 100 mm (ME27)<br><br>25, 47, and 50 mm (ME28 and 29)	0.2 µm<br><br>0.45 µm<br><br>0.6, 0.8, and 1.2 µm<br><br>3 µm	L	
		ii) Capillary Pore Filters				
Polycarbonate Membrane	**Physical Characteristics**<br>• Smooth, thin polycarbonate track Etoh (PCTE) membrane.<br>• Used for particle size classification.<br>• Light gray surface, nearly transparent.<br>• Minimal diffusion of transmitted light.<br>• Low particle collection efficiencies, <70% for some larger pore sizes.<br>• Retains static charge.<br>• Melts at 60 °C.	Whatman Nuclepore Cytiva<br>us.VWR.com, accessed on 1 October, 2022<br>Package of 100	25 mm (black)<br>47 mm (black)<br>25 mm (circles)<br>47 mm (circles)	0.4 µm<br>0.2 µm<br>0.4 µm<br>0.4 µm	L	
		SKC<br>skcinc.com, accessed on 1 October, 2022<br>Package of 100	25 mm	0.8 µm	L	
		Sterlitech Corporation<br>sterlitech.com, accessed on 1 October, 2022<br>Package of 100	13 mm	3 µm	L	

Filter Type	Filter Characteristics and Analysis Methods	Trade Name, Supplier, Website, and Quantity Available	Filter Diameter	Pore Size (Thickness)	Cost US ($)	Comments
	• Moderate flow resistance.<br><br>**Chemical Characteristics**<br>• Low blank levels (made of carbon-based material, so inappropriate for carbon analysis).<br>• High hygroscopicity.<br>• Low blank weight.<br><br>**Compatible Analysis Methods**<br>• Gravimetry, OA, OM, SEM, XRF, PIXE, FTIR, and Raman	Zefon, accessed on 1 October, 2022<br>zefon.com<br>Package of 100	25 and 37 mm	0.2, 0.4, 0.8, and 5 µm	L	
iii) Fibrous Filters						
Cellulose Fiber	**Physical Characteristics**<br>• Thick mat of cellulose fibers or fiber cloth, often called a "paper" fiber filter.<br>• White opaque surface, diffuses transmitted light.<br>• Low particle collection efficiencies, <70% for some variations of this filter.<br>• High mechanical strength<br>• Burns at elevated temperatures (~150 °C, exact temperature depends on	SKC<br>skcinc.com<br>Package of 500	37 mm	NA	L	
		Whatman 41<br>Cytiva<br>us.VWR.com, accessed on 1 October, 2022<br>Package of 100	25, 32, 42.5, 47, 50, 55, 60, 70, 90, 110, 125, 150, 180, 240, and 320 mm diameter, and 20.3 x 25.4 cm^2 and 46 x 57 cm^2	NA	L	• Commonly impregnated with acid or base solution to capture precursor gases such as NO_2, SO_2, NH_3, HNO_3, and organic acid. Typically placed behind the Teflon-membrane or quartz-fiber filters

Filter Type	Filter Characteristics and Analysis Methods	Trade Name, Supplier, Website, and Quantity Available	Filter Diameter	Pore Size (Thickness)	Cost US ($)	Comments
	nature of particle deposit).<br>• Variable flow resistance.<br><br>**Chemical Characteristics**<br>• High purity, low blank levels. Made of carbon-based material, so inappropriate for carbon analysis.<br>• Adsorbs gases, especially water vapor.<br>• Most appropriate for adsorbing gases such as HNO_3, SO_2, NH_3, and NO_2 when impregnated with reactive chemicals.<br>• High hygroscopicity and moisture sensitive.<br>• Low ash content.<br>• High blank weight.<br><br>**Compatible Analysis Methods**<br>• Gravimetry, ICP/AES, ICP/MS, IC, and AC					in the sampling train to capture gas.
Pure Quartz-fiber	**Physical Characteristics**<br>• Mat of pure quartz fibers.<br>• White opaque surface, diffuses transmitted light.<br>• High particle collection efficiencies.<br>• Soft and friable edges	Pallflex Tissuquartz<br>Pall Corporation<br>us.VWR.com, accessed on 1 October, 2022<br>Package of 25 and 100	25, 37, 47, 82.6, 90, 102 mm diameter, and 20.3 cm x 25.4 cm²	(432 μm)	L	• Heat treated.<br>• Most commonly used for aerosol sampling and carbon analysis.
		SKC<br>skcinc.com, accessed on 1	47 mm	1.2 μm	L	

Filter Type	Filter Characteristics and Analysis Methods	Trade Name, Supplier, Website, and Quantity Available	Filter Diameter	Pore Size (Thickness)	Cost US ($)	Comments
	flake in most filter holders.<br>• Stable to ~1000 °C.<br>• Low flow resistance.	October, 2022<br>Package of 100				
	**Chemical Characteristics**<br>• Contains low trace contaminant levels<br>• Passively adsorbs organic vapors. Adsorbs little HNO_3, NO_2, and SO_2.<br>• Low hygroscopicity.<br>• High loading capacity.<br><br>**Compatible Analysis Methods**<br>• ICP/AES, ICP/MS, IC, AC, T, TOR, TMO, TOT, and OA	Whatman<br>Cytiva<br>us.VWR.com, accessed on 1 October, 2022<br>Package of 50<br><br>Package of 25	25, 32, 37, 45, 47, 50, 55, 82, 85, 90, 101.6, 110, 118, and 150 mm diameter,<br><br>and 20.4 cm x 25.4 cm	NA	M to H<br><br>H	

**Filter Type**	**Filter Characteristics and Analysis Methods**	**Trade Name, Supplier, Website, and Quantity Available**	**Filter Diameter**	**Pore Size (Thickness)**	**Cost US ($)**	**Comments**
Mixed quartz-fiber	**Physical Characteristics**<br>• Quartz (SiO_2) fibers with ~5% borosilicate content.<br>• White opaque surface, diffuse transmitted light.<br>• High particle collection efficiencies.<br>• Some batches can melt at ~500 °C. Effects on thermal carbon analysis are unknown.<br>• Becomes brittle when heated.<br>• Low flow resistance.<br>**Chemical characteristics**<br>• Contains large and variable quantities of Na, Al, and Si in all batches. Variable levels of other metals are found in many batches.<br>• Passively adsorbs organic vapors. Adsorbs little HNO_3, NO_2, and SO_2.<br>• Low hygroscopicity.<br>• High blank weight.<br>**Compatible Analysis Methods**<br>• Gravimetry, XRF, PIXE, AA, ICP/AES, ICP/MS for some metals. IC, AC, T, TOR, TMO, and TOT.	Whatman QM/A<br>Cytiva<br>cytivalifesciences.com, accessed on 1 October, 2022<br>Package of 100	25, 32, 37, 47, 50, 55, 82, 85, 90, 101.6, 110, 118, 150 mm diameter, and 20.3 x 25.4 cm^2	(475 µm)	L	• Commonly used in the high volume sampling for TSP (total suspended particles) and PM_{10} (particles with aerodynamic diameter <10 µm).

Filter Type	Filter Characteristics and Analysis Methods	Trade Name, Supplier, Website, and Quantity Available	Filter Diameter	Pore Size (Thickness)	Cost US ($)	Comments
Teflon-coated Glass-Fiber	**Physical Characteristics**<br>• Thick mat of borosilicate reinforced with woven glass cloth and bonded with PTFE.<br>• Glass fiber supporting Teflon is shiny.<br>• High particle collection efficiencies.<br>• Glass melts at ~260°C. Teflon melts at ~60 °C.<br>• Low flow resistance.<br><br>**Chemical Characteristics**<br>• Low blank levels for ions (glass backing and carbon content make it less suitable for elemental and carbon analyses).<br>• Inert to adsorption of HNO_3, NO_2, and SO_2.<br>• Low hygroscopicity.<br>• High blank weight.<br><br>**Compatible Analysis Methods**<br>• Gravimetry, IC, and AC	Pallflex®Emfab™ (TX40HI20ww)<br>Pall Corporation<br>shop.pall.com, accessed on 1 October, 2022<br>Package of 100	25, 37, 47, 70, 81, 85, 90, and 110 mm diameter;20.4 x 25.4 cm²	(178 μm)		• Potential artifact nitrate formation.
Glass-Fiber	**Physical Characteristics**<br>• Borosilicate glass fiber or binder free.<br>• White opaque surface	SKC<br>Type A/E glass fiberskcinc.com<br>Package of 500	25 mm	1 μm (310–350 μm)	L	

Filter Type	Filter Characteristics and Analysis Methods	Trade Name, Supplier, Website, and Quantity Available	Filter Diameter	Pore Size (Thickness)	Cost US ($)	Comments
	diffuses transmitted light.<br>•High particle collection efficiencies.<br>• Melts at ~500 °C.<br>• Low flow resistance<br><br>**Chemical Characteristics**<br>• High blank levels.<br>• Adsorbs HNO_3, NO_2, SO_2, and organic vapors with sulfate and nitrate artifact.<br>• Variable hygroscopicities.<br>• High blank weight.<br><br>**Compatible Analysis Methods**<br>• Gravimetry and OA	Tisch Environmental, Cleavs, OH, USA<br>TE-G653 and TE-EPM2000<br>tisch-env.com, accessed on 2 October, 2022<br>Package of 100	20.3 × 25.4 cm^2	NA	L	

AAS: Atomic Absorption Spectrophotometry; AC: Automated Colorimetry; IC: Ion Chromatography; ICP/AES: Inductively-Coupled Plasma with Atomic Emission Spectrophotometry; ICP/MS: Inductively-Coupled Plasma with Mass Spectrophotometry; INAA: Instrumental Neutron Activation Analysis; NA: not available; OA: Optical Absorption or Light Transmission (b_{abs}); OM: Optical Microscopy; PIXE: Proton-Induced X-Ray Emissions; SEM: Scanning Electron Microscopy; T: Thermal Carbon Analysis; TEM: Transmission Electron Microscopy; TMO: Thermal Manganese Oxidation Carbon Analysis; TOR: Thermal/Optical Reflectance Carbon Analysis; TOT: Thermal/Optical Transmission Carbon Analysis; XRD: X-Ray Diffraction; XRF: X-Ray Fluorescence; FTIR: Fourier Transform Infrared Spectrometry; Raman: Raman Spectrometry; * Cost; Low: less than $300 per 100 filters; Medium: between $301–500 per 100 filters; High: more than $501 per 100 filters

Table S2. Mass concentration under uncontrolled and controlled temperature and humidity environments [126].

Filter Type (Manufacturer)	Uncontrolled Temperature and Humidity (Average ± Std. Dev.)	CV[a]	Controlled Temperature[b] and Humidity (Average ± Std. Dev.)	CV[a]
Teflon membrane filter (Omega)	244.371 ± 0.0047 mg	0.0019%	244.370 ± 0.0049 mg	0.0020%
PVC membrane filter (Omega)	13.259 ± 0.0021 mg	0.0160%	13.257 ± 0.0023 mg	0.0174%
MCE membrane filter (Gelman)	41.582 ±0.1132 mg	0.2722%	41.435 ± 0.0431 mg	0.104%
Glass-fiber filter (Whatman)	87.933 ± 0.0628 mg	0.0714%	87.844 ± 0.0118 mg	0.0134%

[a]CV: Coefficient of variation is the ratio of standard deviation to the mean expressed as percentage. Tsai et al (2002) define the CV as relative deviation. [b]Controlled Experimental Chamber condition: temperature of 22–26 °C and relative humidity 39.5–41%. Uncontrolled Experimental Chamber condition: 24–28.5 °C and relative humidity 40–65%. Manufacturers are Omega Specialty Intrument Co., Chelmsford, MA, USA, Gelman Sciences, Inc. Ann Arbor, MI, USA, Whatman, Inc., Florham Park, NJ, USA.

Table S3. Major infrared bands of the principal polymorphs in crystalline silica [140].

Polymorph	Primary Absorbance	Secondary Absorbance
α-quartz	780 and 799 cm^{-1}	695 cm^{-1}
Cristobalite	621 cm^{-1}	796 cm^{-1}
Tridymite	567 cm^{-1}	789 cm^{-1}

S1. Supplemental Figures

Figure S1. Historical examples of mine safety equipment.

Figure S2. Coal Mine Dust Personal Sampler Unit (CMDPSU).

Figure S3. Personal Dust Monitor (PDM3700).

Figure S4. Scanning electron microscopic images for different filters.

Figure S5. Penetration as a function of particle diameter for different filters.

Figure S6. Pressure drop as a function of sampling flow rate for silver membrane filters.

Figure S7. Examples of filter deposits.

Figure S8. FTIR spectra for blank Teflon coated glass-fiber filters.

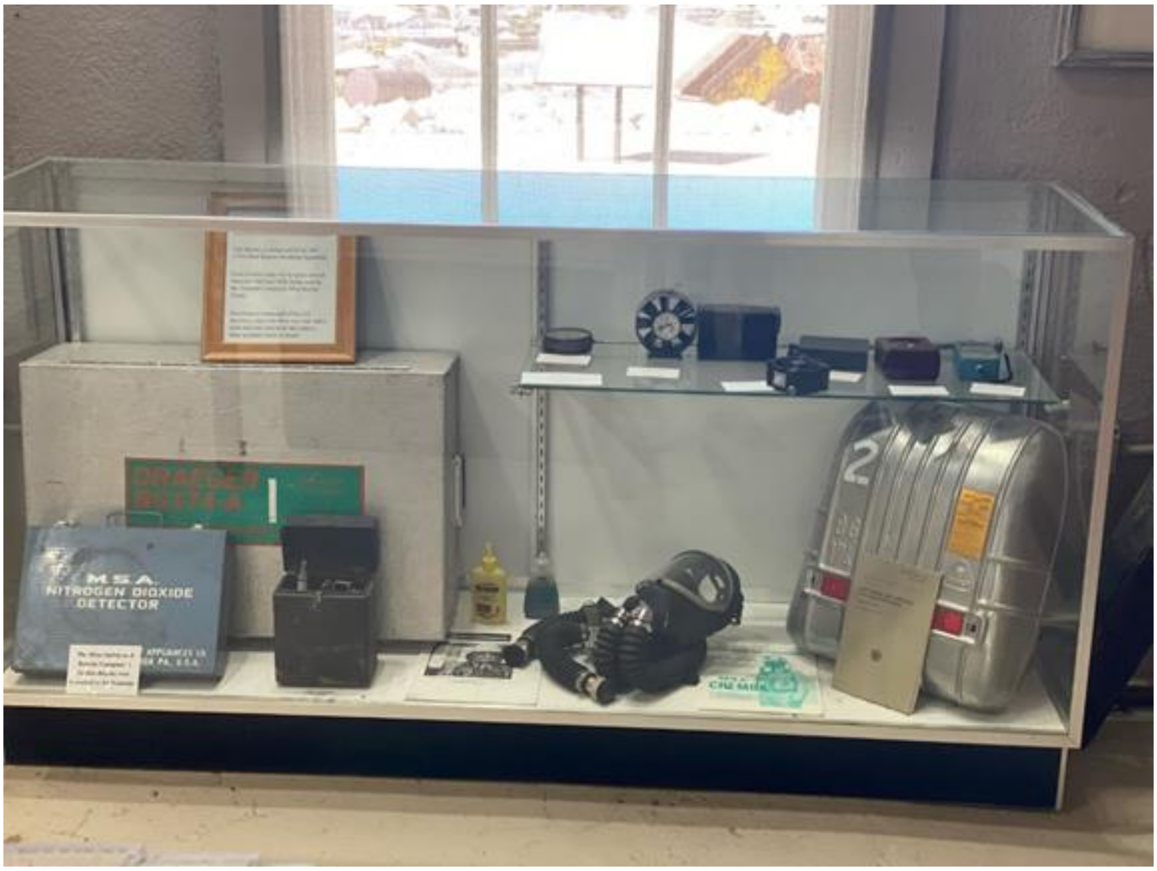

Figure S1. Historical examples of mine safety equipment used in the Tonopah Belmont Gold Mine, Nevada.

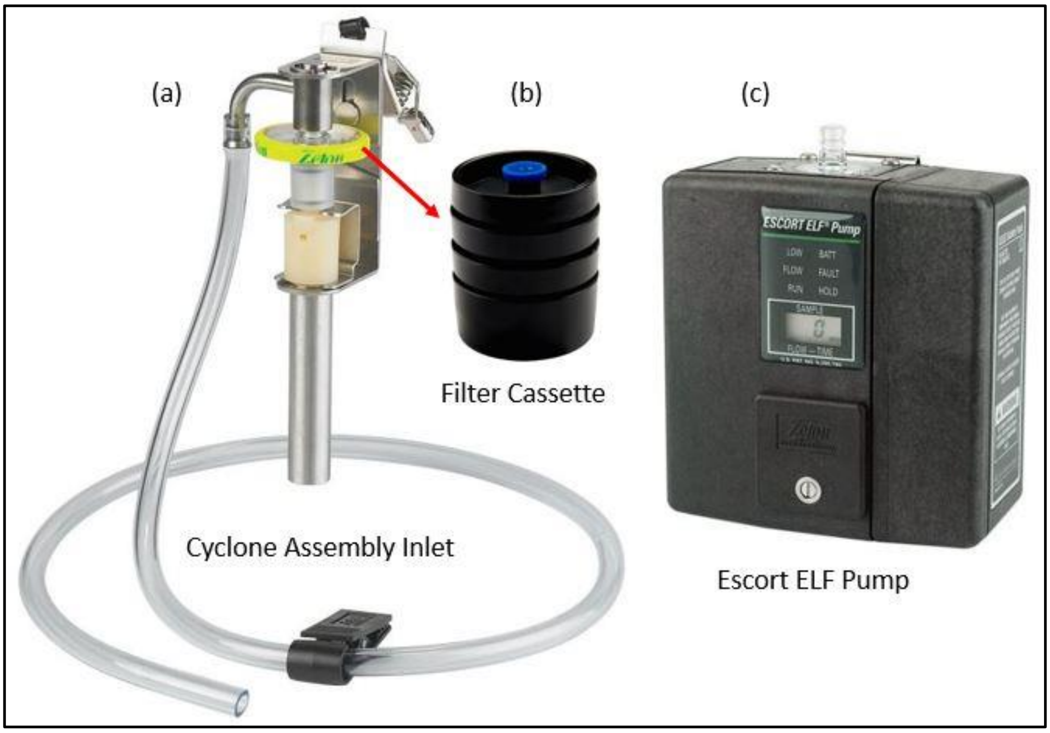

Figure S2. Coal Mine Dust Personal Sampler Unit (CMDPSU, www.zefon.com (accessed on 1 October, 2022), including: **a**) cyclone assembly with single stage filter folder; **b**) 4 piece conductive cassette housing; and **c**) personal sampling pump.

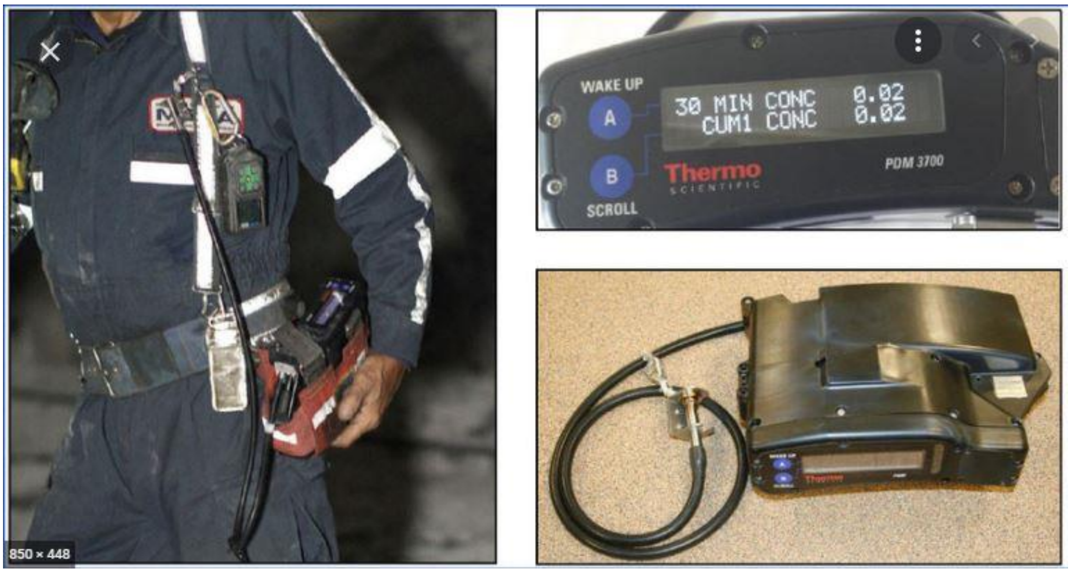

Figure S3. A wearable Personal Dust Monitor (PDM3700, www.thermofisher.com (accessed on 1 October, 2022), for acquiring respirable coal mine dust (RCMD) mass concentrations. Reproduced with permission from Thermo Scientific.

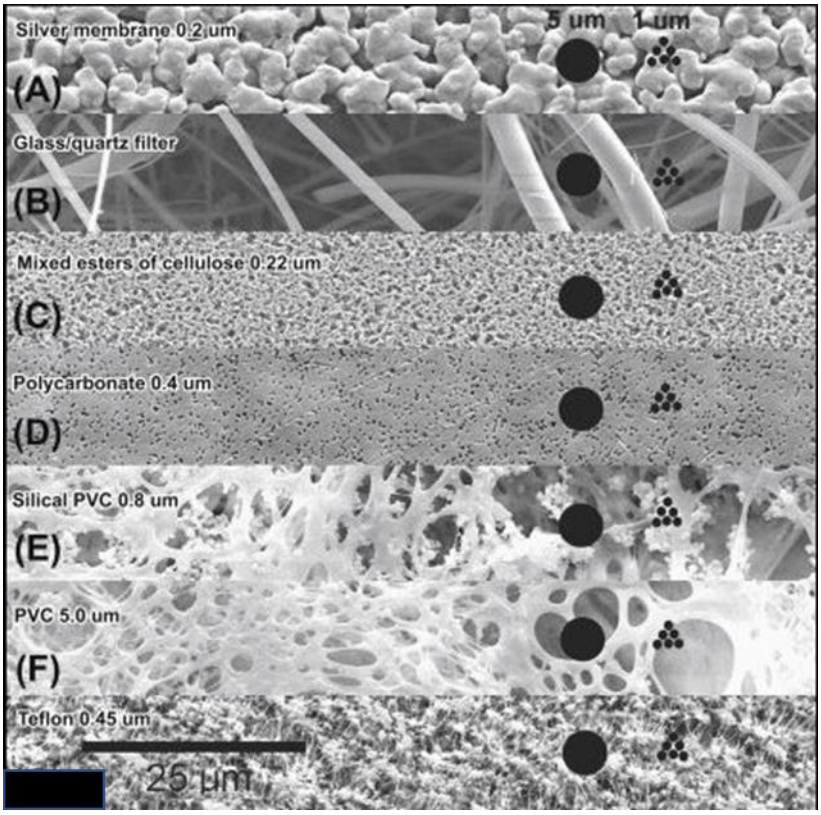

Figure S4. Scanning electron microscopic images for: **A**) 0.2 μm silver membrane; **B**) glass/quartz filter; **C**) 0.22 μm mixed cellulose ester membrane; **D**) 0.4 μm polycarbonate; **E**) 0.8 μm Silical PVC; **F**) 5.0 μm PVC; and **G**) 0.45 μm Teflon membrane filters [67]. Reprinted with permission from Academic Press.

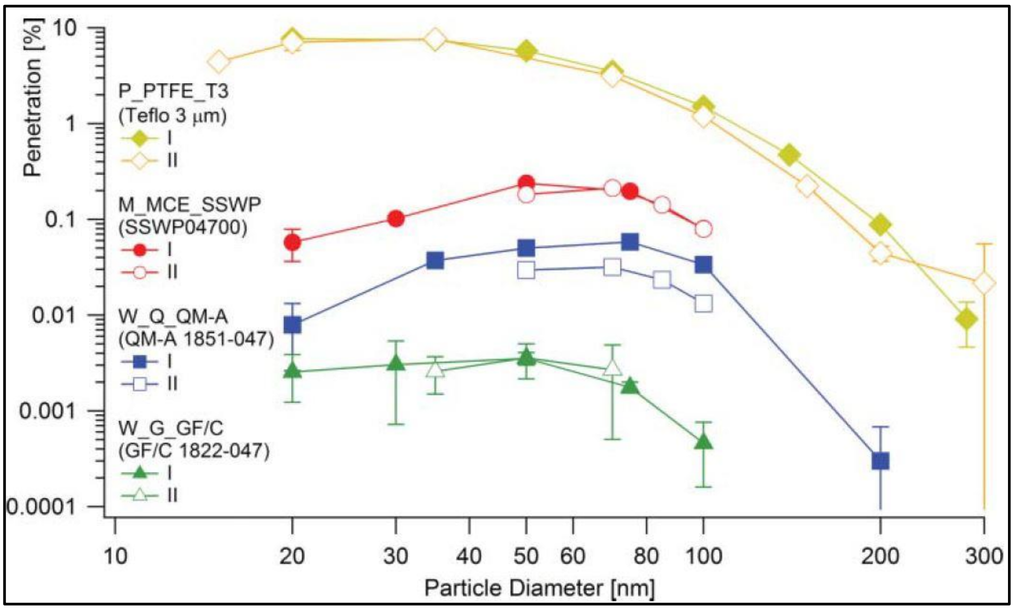

Figure S5. Penetration as a function of particle diameter for polytetrafluoroethylene (PTFE, 3 µm), mixed cellulose esters (MCE), mixed quartz-fiber (QMA), and glass-fiber (GF) filters. Test with different batch of filters than those shown in Figures 2 and 3 [106].

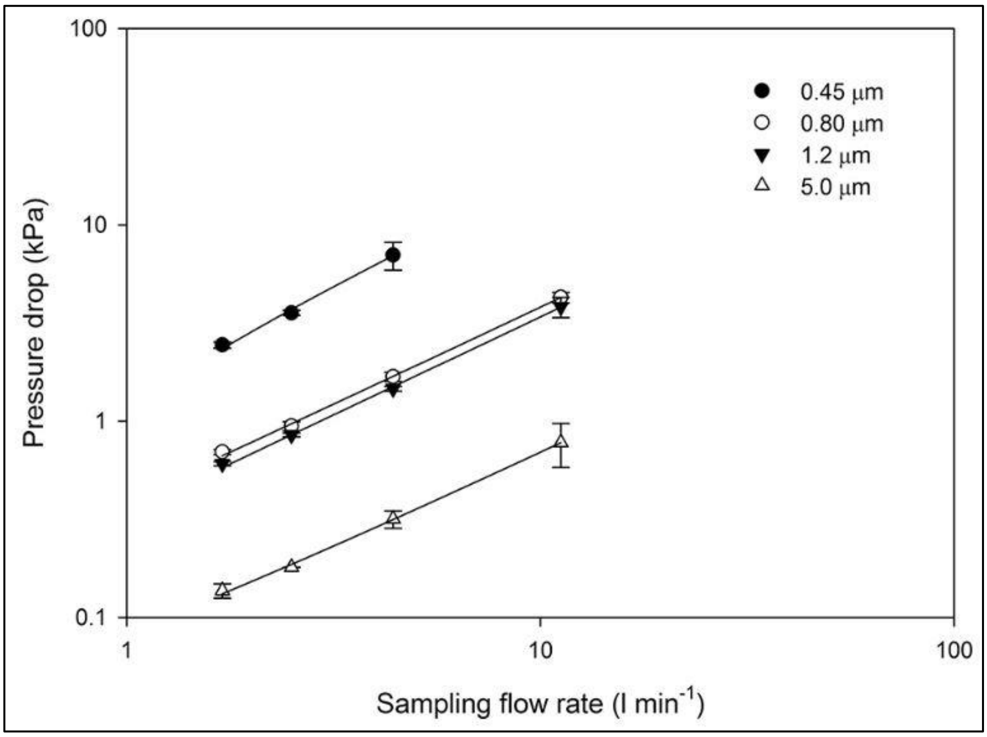

Figure S6. Pressure drop as a function of sampling flow rate for silver membrane filters with pore size diameters of 0.45, 0.8, 1.2 and 5 µm [133].

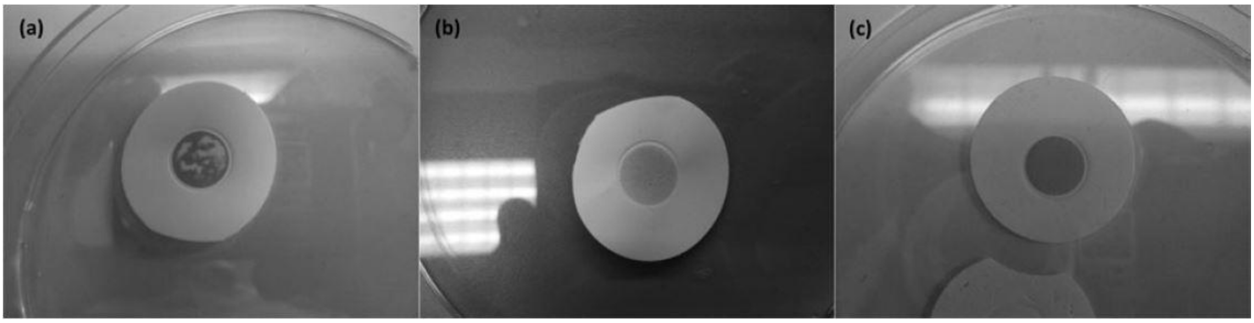

Figure S7. Examples of: **a**) uneven particle deposition on Nylon filter deposited using the NIOSH 7603 method; **b**) even particle deposition on a polypropylene filter deposited using MSHA method P-7; and **c**) even particle deposition on a PVC filter deposited using NIOSH 7603 method [52].

a) b)

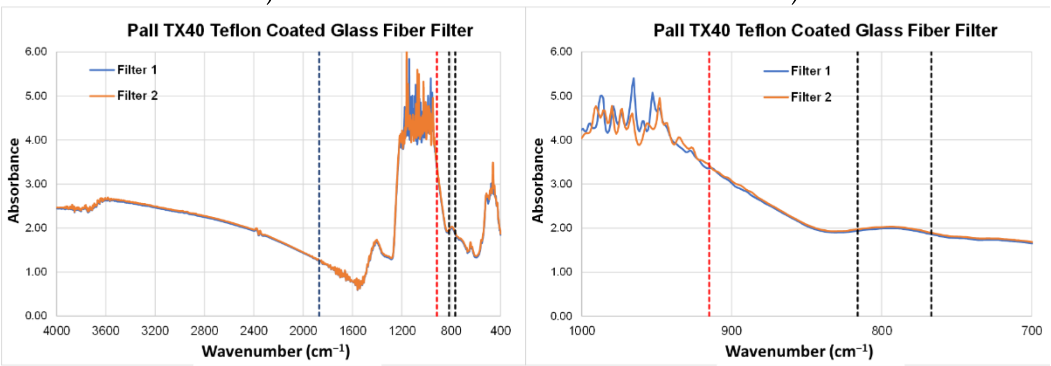

Figure S8. FTIR spectra for blank Teflon coated glass-fiber filters (TX40HI20, Pall Corporation) for: **a**) full spectra and **b**) zoom-in spectra at 700–1000 cm^{-1}. The vertical black dashed lines indicate crystalline silica absorption peaks at 767–816 and 1870 cm^{-1}. Kaolinite also absorbs at 767–816 cm^{-1}, causing interference. Additionally, kaolinite absorbs at 915 cm^{-1} (red dash line).
